# Metabolic switch: lymphocytes and the onset of T-cell-mediated autoimmunity

**DOI:** 10.1038/s41423-026-01460-5

**Published:** 2026-08-24

**Authors:** Andisheh Mosaffa Jahromi, Fatemeh Mirzaei, Sara Mirzazadeh, Seppo Meri, Dieter Kabelitz, Kurosh Kalantar

**Affiliations:** 1 Department of Immunology, School of Medicine, Shiraz University of Medical Sciences, Shiraz, Iran; 2 Department of Bacteriology & Immunology and the Translational Immunology Research Program (TRIMM), University of Helsinki, Helsinki, Finland; 3 Institute of Immunology, Christian Albrechts University of Kiel and University Hospital Schleswig, Holstein Campus Kiel, Kiel, Germany; 4 Autoimmune Diseases Research Centre, Shiraz University of Medical Sciences, Shiraz, Iran

**Keywords:** B cells, T cells, Immunometabolism, Autoimmunity, Immunology, Cell biology

## Abstract

T cell-mediated autoimmune diseases, including multiple sclerosis (MS), rheumatoid arthritis (RA), and type 1 diabetes (T1D), are being increasingly recognized as disorders driven not only by immune dysregulation but also by profound metabolic reprogramming in lymphocytes. Emerging evidence from the field of immunometabolism reveals that altering the balance between oxidative phosphorylation (OXPHOS) and aerobic glycolysis, along with enhancing fatty acid synthesis and dysregulated glutamine metabolism, critically shapes lymphocyte activation, differentiation, and pathogenicity. Here, we review the metabolic pathways that regulate T cells and B cells. We discuss how changes in glucose, lipid, and mitochondrial metabolism influence immune responses that lead to chronic inflammation and autoimmunity in MS, RA, and T1D. Interestingly, similar immunometabolic changes, such as increased glycolysis, mitochondrial dysfunction, and mTOR signaling, have been identified in another autoimmune disorder, systemic lupus erythematosus (SLE). Connecting metabolic dysregulation to immune tolerance failure, this review highlights immunometabolism as a key mechanism in autoimmunity. Immunometabolic pathways represent a new avenue for precision immunotherapy, although challenges persist in targeting cells specifically without systemic toxicity. Understanding these metabolic adaptations and epigenetic–metabolic crosstalk will be essential for translating these insights into next-generation therapies.

## Introduction

The adaptive immune system consists of T and B lymphocytes. It plays a central role in protecting the host from pathogens and tumor cells. However, this strong defense system can also become harmful when it mistakenly attacks the body’s own healthy tissues. This can lead to autoimmunity and possibly irreversible damage to vital organs [1]. To prevent these harmful immune responses, the immune system uses central and peripheral tolerance mechanisms to eliminate or control self-reactive T and B cells.

Autoimmune responses typically arise through two major mechanisms: T cell-mediated and antibody-mediated pathways. T cell-mediated autoimmunity contributes significantly to the pathogenesis of several autoimmune diseases, including multiple sclerosis (MS), type 1 diabetes (T1D), rheumatoid arthritis (RA) and  systemic lupus erythematosus (SLE). Key mechanisms involved in these conditions include direct tissue damage by cytotoxic CD8⁺ T cells, the secretion of proinflammatory cytokines by CD4⁺ T helper subsets, and dysregulated interactions between T cells and antigen-presenting cells (APCs) or B cells, which collectively amplify the autoimmune cascade [2–4].

In recent years, the field of immunometabolism has emerged as a critical area of research, revealing how immune cell functions are tightly regulated by metabolic pathways. It is now well established that distinct metabolic programs underlie the differentiation, activation, and effector functions of lymphocytes. Moreover, increasing evidence suggests that metabolic dysregulation can drive inappropriate immune activation and contribute to the onset and progression of autoimmune diseases [5].

Although various studies have highlighted metabolic alterations in different immune cells across multiple autoimmune disorders, a comprehensive analysis of metabolic reprogramming in lymphocytes detailing how these alterations specifically affect lymphocyte functions in various T cell-mediated autoimmune diseases remains undefined. In this review, we first characterize the metabolic pathways associated with lymphocytes during the immune response. Next, we review evidence on how metabolic dysregulation modulates lymphocyte behavior in T cell-driven autoimmune pathologies. Notably, we connect immunometabolism to the development of these diseases and highlight potential molecular targets for future therapeutic interventions.

## Main metabolic pathways in immune cells

Metabolism encompasses a series of cellular processes that regulate chemical reactions, enabling the conversion of nutrients into energy. The primary dietary components, including carbohydrates, fats, and proteins, serve as crucial energy sources for the body. To meet cellular energy demands, several interconnected metabolic pathways play essential roles: glycolysis, the tricarboxylic acid (TCA) cycle, oxidative phosphorylation (OXPHOS), and fatty acid metabolism [6].

Glycolysis takes place in the cytosol [6], whereas the TCA cycle and OXPHOS occur within the mitochondria. This process begins with glucose uptake from the extracellular environment via glucose transporters such as glucose transporter 1 (GLUT1). Once glucose enters the cell, it is phosphorylated by hexokinases to form glucose-6-phosphate (G6P). During glycolysis, G6P is further broken down into pyruvate, generating two adenosine triphosphate (ATP) molecules and reducing nicotinamide adenine dinucleotide (NAD⁺) to NADH [7]. Furthermore, G6P can also enter the pentose phosphate pathway (PPP), a branch of glycolysis that generates nicotinamide adenine dinucleotide phosphate (NADPH). NADPH acts as a reducing agent and a precursor for the ribose sugars needed for nucleotide synthesis [6]. Another important metabolic pathway, the TCA cycle, also known as the Krebs cycle, occurs in the mitochondrial matrix. It plays a crucial role in the metabolism of various fuels to generate reduced coenzymes such as NADH and flavin adenine dinucleotide (FADH₂), which drive ATP production via the electron transport chain (ETC) [8]. Accordingly, OXPHOS transfers electrons from NADH and FADH₂ through a series of mitochondrial carriers, resulting in ATP production [9, 10].

**Fig. 1:**
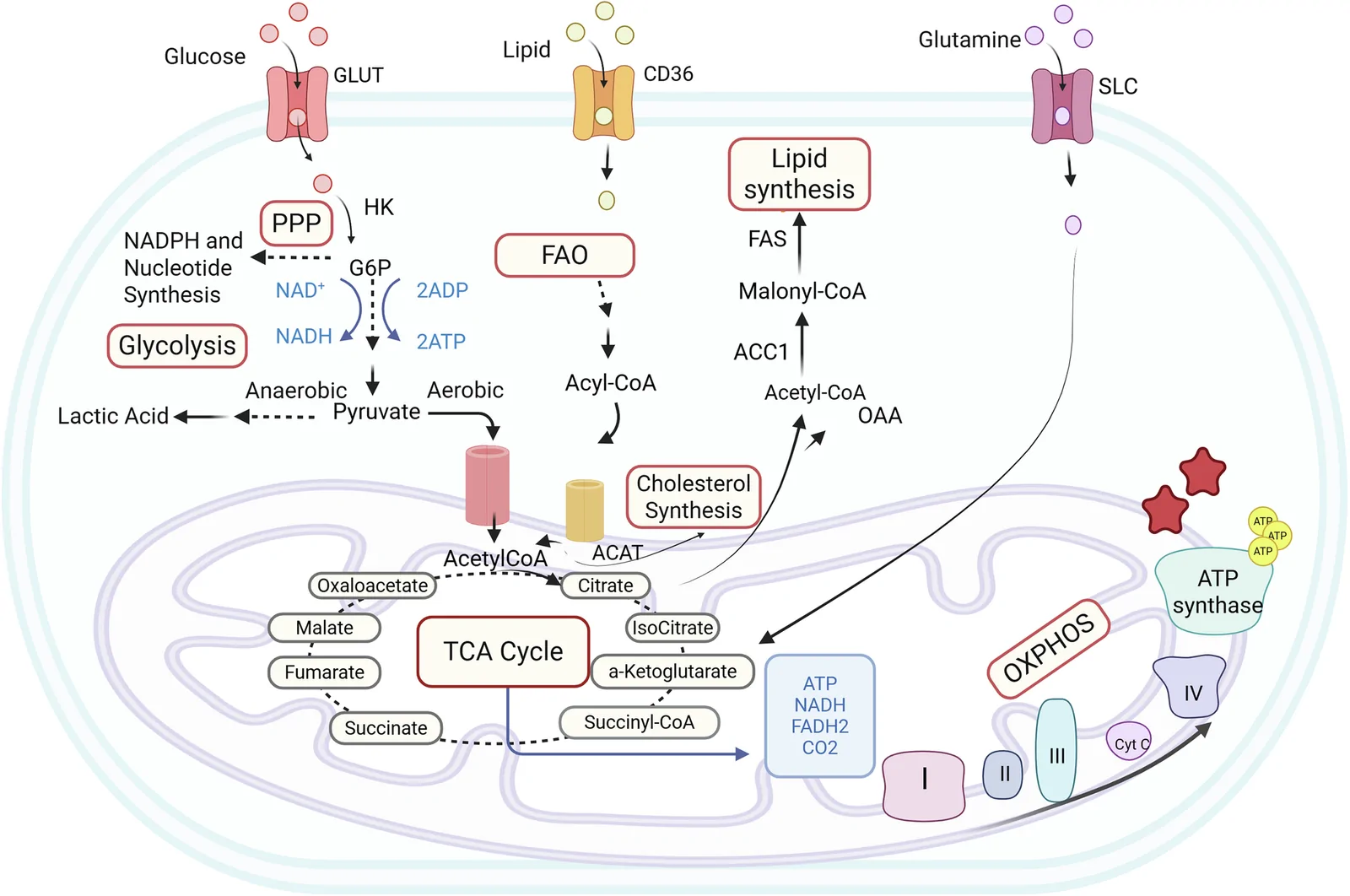
Key metabolic pathways for generating energy in cells. Glucose is converted into pyruvate through the glycolytic pathway, generating ATP and NADH. Pyruvate is then converted to acetyl-CoA, which enters the mitochondrial TCA cycle and is further metabolized to produce NADH and FADH₂, which are used for ATP production via OXPHOS. ATP can also be produced from fatty acids through beta-oxidation. Additionally, glutamine can serve as a source of ATP through glutaminolysis. Abbreviations: FAO, fatty acid oxidation; OXPHOS, oxidative phosphorylation; PPP, pentose phosphate pathway; TCA, tricarboxylic acid. The figure was created with BioRender software

In fatty acid metabolism, acetyl coenzyme A (acetyl-CoA) derived from glycolysis can be utilized for de novo fatty acid or cholesterol synthesis. In the mitochondria, it combines with oxaloacetate to form citrate, which is transported to the cytosol and converted back into acetyl-CoA. Acetyl-CoA is then carboxylated by acetyl-CoA carboxylase 1 (ACC1) to produce malonyl-CoA, which fatty acid synthase (FASN) uses to extend acyl chains, ultimately forming palmitate. Alternatively, two acetyl-CoA molecules can initiate cholesterol synthesis via acetoacetyl-CoA thiolase (ACAT). While fatty acid synthesis is anabolic, fatty acids can also undergo β-oxidation for energy production. This requires their transport into mitochondria, where they are broken down into acetyl-CoA, generating NADH and FADH₂ to fuel the ETC [11]. The key metabolic pathways that are important for generating energy in immune cells are summarized in Fig. 1.

### Overview of T cell metabolism

T cells exhibit metabolic plasticity on the basis of their differentiation state (naïve, activated, or memory) and subset specificity (CD4^+^ T helper (Th) subsets, CD8^+ ^ cytotoxic T lymphocytes (CTLs) or regulatory T cells (Tregs)), and this correlates with the differential use of metabolic pathways. Naïve T cells primarily depend on OXPHOS to maintain survival while in a resting state. This catabolic metabolism supports their quiescent state, maintaining cell survival without rapid proliferation [11, 12]. Upon activation, naïve T cells differentiate into effector T cells, such as Th1, Th2, Th17 and CTLs. Activated T cells undergo rapid proliferation and differentiation, which require elevated energy substrates. They meet these energy demands through metabolic reprogramming, which is characterized by increased glycolysis, fatty acid synthesis and glutaminolysis [13, 14]. Mechanistically, the upregulation of the transcription factor c-Myc plays a crucial role in enhancing activation-induced glycolysis. c-Myc transcriptionally regulates several glycolytic genes, including Glut1, the primary glucose transporter in lymphocytes. While Glut1 is minimally expressed on the surface of naïve T cells, its levels increase rapidly on the plasma membrane following activation. Therefore, the induction of Glut1 is regarded as a vital step in promoting T cell activation and responsiveness [15, 16].

In addition, fatty acid production plays a significant role in the functionality of effector T cells. Although activated T cells can take up and utilize fatty acids from their environment, there appears to be an inherent requirement for de novo synthesized fatty acids. Studies have shown that the inhibition of ACC1, a key enzyme in fatty acid synthesis, restricts the differentiation of Th17 cells while promoting the development of Tregs [17, 18].

Importantly, activated T cells also utilize glutamine to support their metabolic processes, serving as both a nitrogen source and an anaplerotic substrate [19]. Compared with Th1 and Tregs, Th17 cells have higher rates of glutaminolysis and rely heavily on this pathway for functional differentiation. Th1 cells do not require glutaminase 1 (GLS1), the key enzyme in glutaminolysis, for effector cytokine production. However, compared with wild-type Th17 cells, GLS1-deficient Th17 cells produce significantly lower levels of IL-17. In contrast, inhibiting GLS1 activity increases Th1 cytokine production and T-bet expression [20].

Tregs are a distinct subset of CD4⁺ T cells but can also be induced under specific conditions from those of naïve CD4^+^ T cells (induced Tregs, iTregs). Like naïve T cells, Tregs are less dependent on glycolysis and rely more on fatty acid oxidation (FAO) and OXPHOS. This metabolic difference implies that distinct energy pathways underlie the specialized suppressive functions of Tregs, highlighting the need for further investigation [11, 21].

Following antigen-specific activation, many effector T cells undergo programmed cell death. However, a subset survives long-term, developing into memory T cells capable of responding more quickly to antigenic challenge, such as reinfections or tumor recurrences. Effector and memory T cells exhibit distinct mitochondrial adaptations aligned with their metabolic needs. Effector T cells reposition mitochondria toward immune synapses to increase calcium influx. In contrast, memory T cells reorganize mitochondria to associate with the endoplasmic reticulum (ER), which in turn increases aerobic glucose metabolism and IFN-γ production during secondary responses. Memory T cells primarily rely on OXPHOS for long-term persistence and possess increased spare respiratory capacity (SRC), mitochondrial content, and expression of key ETC proteins.

**Fig. 2:**
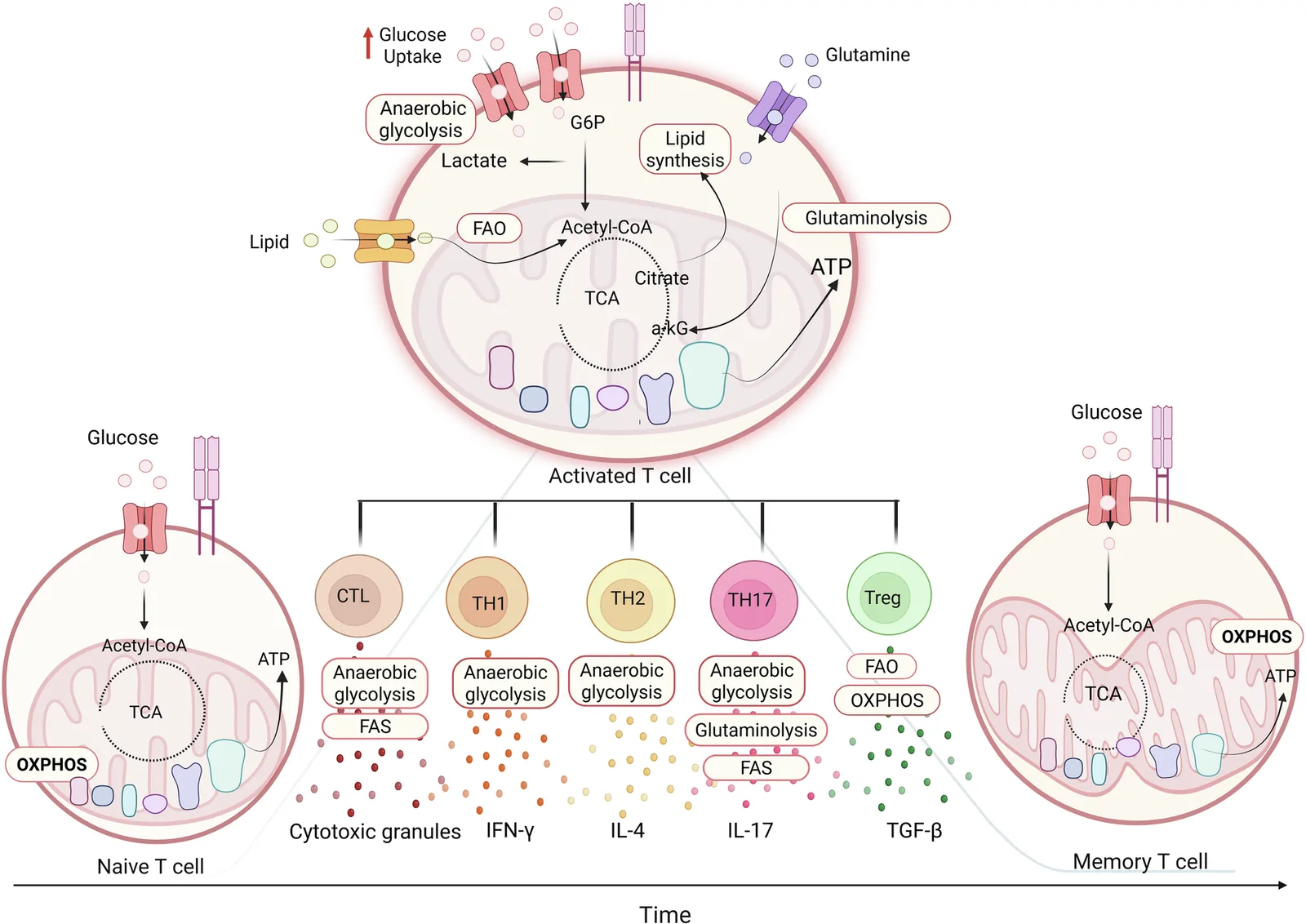
Metabolic alterations during T cell differentiation. Naïve T cells primarily use OXPHOS for survival (left). Rapid proliferation and cytokine production in activated effector T cells depend on anaerobic glycolysis and glutaminolysis (middle). Memory T cells also rely on OXPHOS for long-term persistence and meet their metabolic needs (right). Abbreviations: FAS, fatty acid synthesis. The figure was created with BioRender software

Furthermore, CD8⁺ memory T cells exhibit elongated mitochondria because of fusion events, which increase NADH oxidation [11, 22]. When these memory T cells reencounter the same antigen, they rapidly activate effector functions to eliminate the threat. Effector T cells derived from memory T cells, as opposed to those derived from naïve precursors, are more efficient at producing cytolytic effector proteins. This increased efficiency is due to better coordination of glycolytic enzymes and mitochondrial function, allowing rapid glucose utilization during antigen re-exposure [23]. The metabolic alterations during T cell differentiation are summarized in Fig. 2.

### Overview of B cell metabolism

Metabolism has been increasingly recognized as a crucial factor influencing B cell function, similar to its role in T cells. Naïve B cells use mainly OXPHOS and FAO to produce energy. They maintain a low metabolic rate to conserve resources, remaining poised for activation upon antigen encounter. Upon activation, B cells shift toward increased glycolysis and glucose uptake to support rapid proliferation and antibody production. Glucose-derived pyruvate generated through glycolysis enhances OXPHOS to meet energy demands. Unlike activated T cells, which rely predominantly on glycolysis, activated B cells sustain higher OXPHOS levels to support antibody glycosylation, increase mitochondrial mass, and upregulate the expression of mitochondrial respiration genes.

In germinal centers (GCs), B cells undergo dynamic transitions between the dark zone (DZ), characterized by rapid proliferation and somatic hypermutation, and the light zone (LZ), where they enter a transient quiescent state to compete for T follicular helper (Tfh) cell signals [24]. Despite their proliferative state in the DZ, GC B cells predominantly rely on FAO rather than glycolysis, a metabolic profile atypical of rapidly dividing cells [25]. This reliance on FAO supports energy demands while maintaining low glycolytic activity, likely because of nutrient constraints within the GC microenvironment.

In the LZ, limited oxygen and nutrient availability activate transcription factors such as hypoxia-inducible factor 1-alpha (HIF-1α), AMP-activated protein kinase (AMPK), and glycogen synthase kinase-3 (GSK3). These factors suppress anabolic pathways, including phosphatidylinositol 3-kinase (PI3K) and mammalian target of rapamycin (mTOR), while promoting c-Myc degradation and shifting metabolism toward catabolism [26]. HIF-1α enhances glycolysis but restricts pyruvate entry into the TCA cycle, limiting OXPHOS [27]. B cells that successfully capture antigen and receive help from Tfh cells upregulate c-Myc, triggering a glycolytic burst and mitochondrial biogenesis. This metabolic shift supports renewed proliferation and enables re-entry into the DZ for further affinity maturation, highlighting the metabolic plasticity of GC B cells in adapting to variable nutrient signals [28, 29].

Upon differentiation into plasma cells (PCs), B cells undergo profound metabolic reprogramming to meet the bioenergetic and biosynthetic demands of antibody production. PCs exhibit high OXPHOS and de novo fatty acid synthesis, which support the expansion of the ER and Golgi complex for efficient antibody synthesis and secretion. Transcription factors such as B lymphocyte-induced maturation protein 1 (BLIMP1), mTOR complex 1 (mTORC1), and c-Myc orchestrate these adaptations by enhancing mitochondrial remodeling and anabolic capacity [26]. This metabolic state ensures that PCs can sustain the high energy demands of their secretory function.

Memory B cells, formed in GC reactions, display a balanced metabolism that combines the properties of both naïve and activated B cells. They utilize both OXPHOS and glycolysis, enabling a state of metabolic priming that supports rapid reactivation upon antigen re-exposure [30]. This flexibility supports their ability to mount rapid and robust immune responses, contributing to long-lasting immunological memory.

**Fig. 3:**
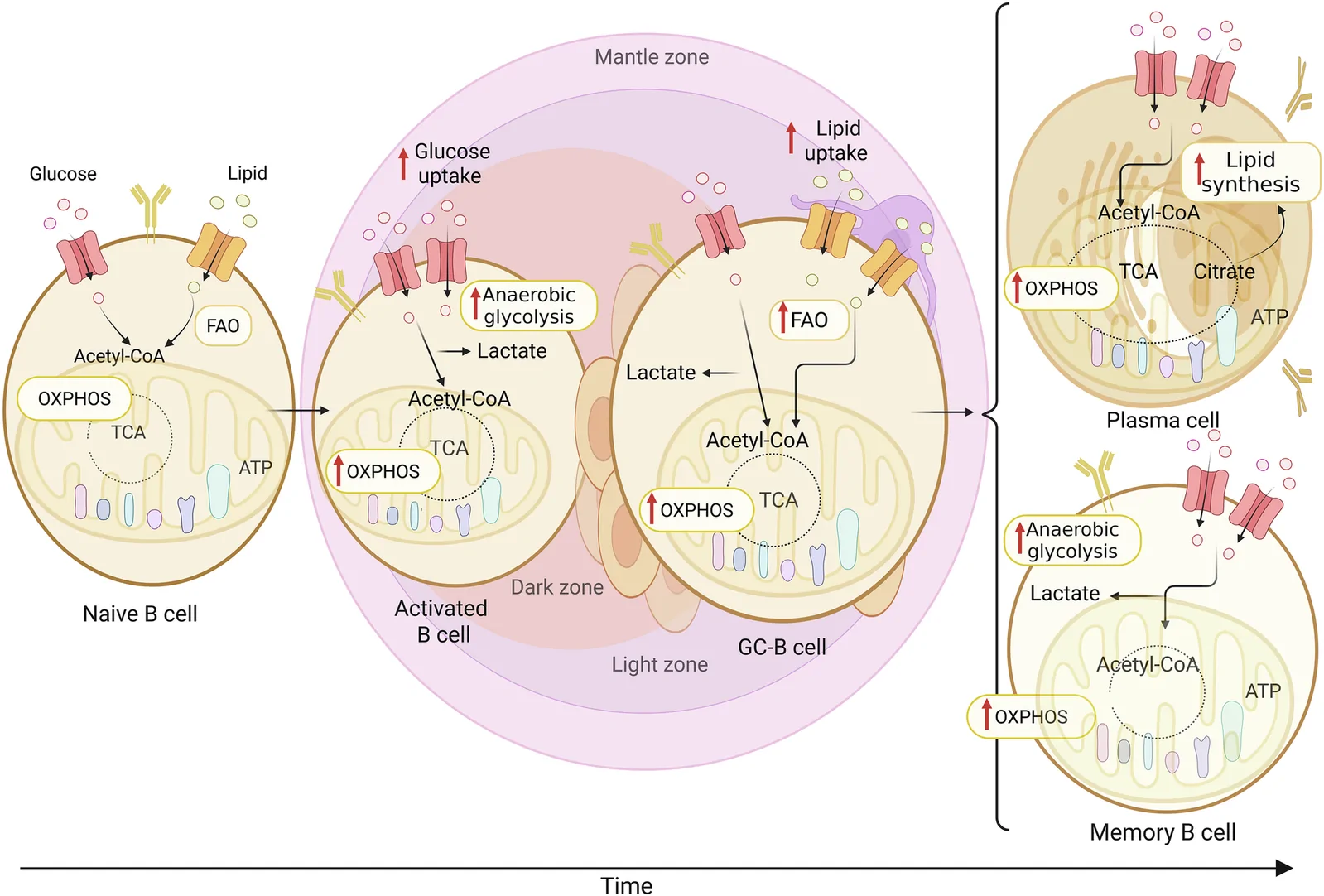
Metabolic alterations during B-cell activation. Naïve B cells primarily rely on oxidative phosphorylation and fatty acid oxidation (left). Upon activation, B cells depend on increased glycolysis to meet their energy demands. Germinal center B cells also rely on fatty acid oxidation and OXPHOS (middle). Once differentiated into plasma cells, they use de novo fatty acid synthesis and oxidative phosphorylation to generate large amounts of antibodies (upper right). Memory B cells use both glycolysis and oxidative phosphorylation to quickly respond to antigens (lower right). Abbreviations: GC, germinal center. The figure was created with BioRender software

In summary, the metabolic plasticity of B cells, including FAO in GC B cells, high OXPHOS in PCs, and balanced metabolism in memory B cells, enables their functional diversity and adaptability within the immune system. These metabolic shifts are tightly regulated by environmental cues and transcription factors, ensuring effective humoral immunity. The metabolic alterations during B cell differentiation are summarized in Fig. 3.

In the following sections, we discuss how metabolic reprogramming in T cells and B cells potentially contributes to disease progression in three types of (mainly) T cell-driven autoimmune diseases.

## Multiple sclerosis

MS is a prototypical T cell-mediated autoimmune disease of the central nervous system (CNS) triggered by an interplay between genetic susceptibility and environmental factors. These risk factors contribute to abnormal activation of the immune system in the periphery, which then infiltrates the CNS, leading to inflammation, neurodegeneration, and destruction of the myelin sheath [31].

### Dysregulation of CD4^+^ T cell metabolism in MS

The primary immune cells in the CNS responsible for myelin damage and axon loss are autoreactive CD4^+^ T cells and macrophages. In MS, numerous Th1 and pathogenic Th17 cells have been found in the cerebrospinal fluid (CSF). These myelin-specific CD4^+^ T cells release proinflammatory cytokines such as IL-17, IFN-γ, and GM-CSF, supporting CNS inflammation and exacerbating demyelinating lesions [31].

**Fig. 4:**
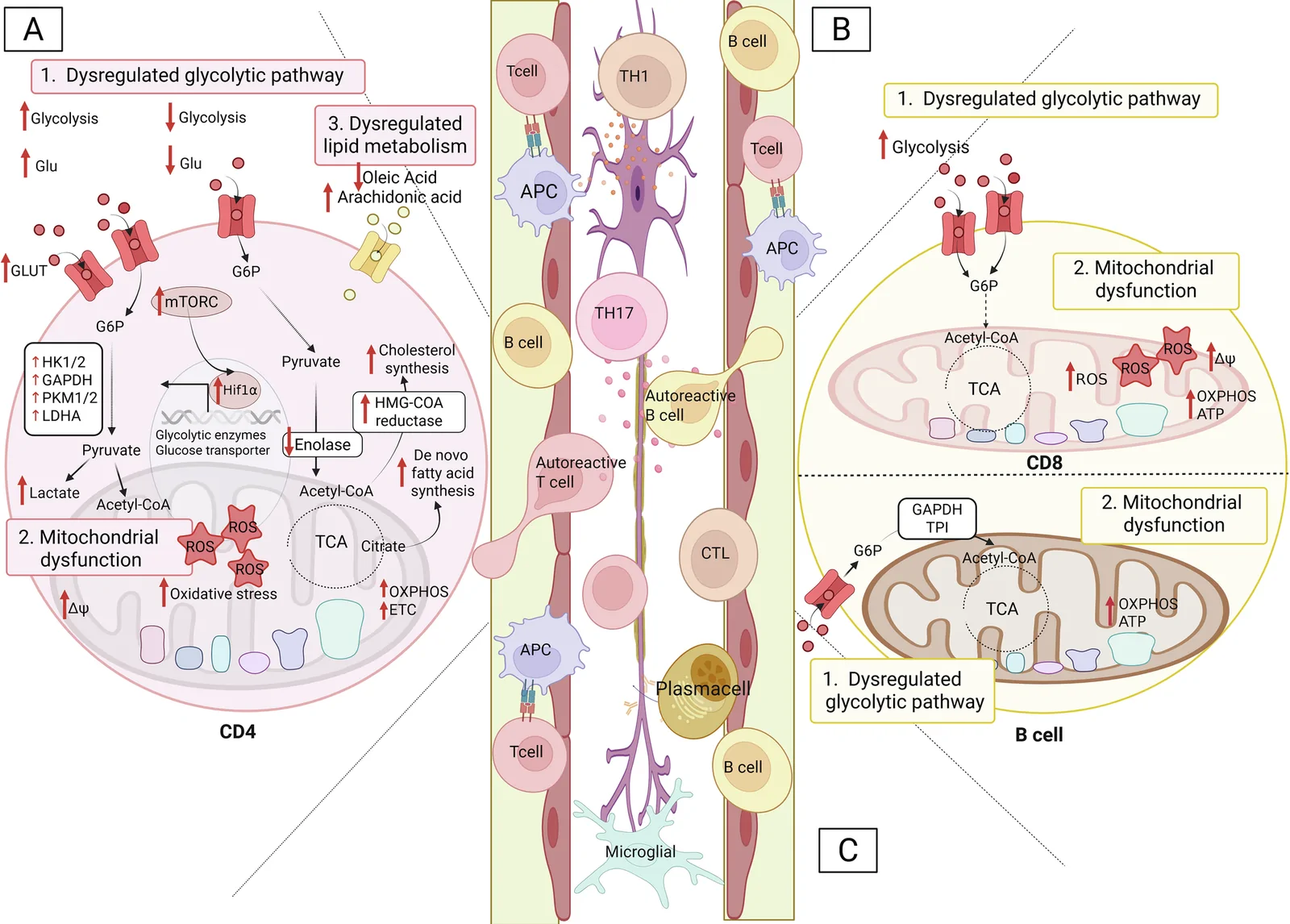
Metabolic alterations in T cells and B cells in multiple sclerosis. **A** Autoreactive CD4^+^ T cells exhibit elevated mTORC1 signaling and HIF-1α expression, driving a metabolic shift toward glycolysis, which is essential for Th1 and Th17 differentiation. Impairments in glycolytic pathways also compromise inducible regulatory T cell (iTreg) induction. Mitochondrial dysfunction, characterized by decreased mitochondrial transmembrane potential, reduced superoxide dismutase (SOD) and glutathione peroxidase (GPX) activity, enhanced reactive oxygen species (ROS) production, and electron transport chain (ETC) activation, promotes autoreactive CD4^+^ T cell survival, oxidative stress accumulation, and Th17 polarization. Dysregulation of lipid metabolism further alters lipid composition, thereby facilitating Th1 and Th17 responses and enabling CD4^+^ T cell infiltration into the central nervous system (CNS). **B** Autoreactive CD8^+^ T cells similarly undergo glycolytic metabolic reprogramming, which augments the release of cytotoxic granules. Mitochondrial defects in these cells also contribute to increased ROS production. **C** B cells in MS rely on glycolytic metabolism, and enhanced oxidative phosphorylation (OXPHOS) in these cells potentiates their antigen-presenting capacity and promotes inflammatory cytokine production

One of the most critical factors determining T cell function and differentiation under both physiological and pathological conditions is the alteration of metabolic pathways. Although the link between T cell metabolic changes and MS development remains incompletely understood, several key metabolic alterations have been identified, as summarized in Fig. 4A.

#### Dysregulated glycolytic pathway in CD4^+^ T cells in MS

Alterations in glucose metabolism are particularly prominent in T lymphocytes from both MS patients and experimental autoimmune encephalomyelitis (EAE) mouse models. In EAE mice, increased glycolytic activity in CD4^+^ T cells plays a major role in disease progression. Compared with that in healthy mice, prolonged activation of mTORC1 signaling in CD4^+^ T cells leads to increased expression of the transcription factor HIF-1α, an essential mediator that dramatically increases the expression of enzymes related to the glycolysis pathway, in EAE mice [32]. The mRNA expression of key glycolytic enzymes and transporters such as GLUT1, hexokinase 1/2 (HK1/2), glyceraldehyde 3-phosphate dehydrogenase (GAPDH), pyruvate kinase isoform M1/2 (PKM1/2), and lactate dehydrogenase A (LDHA) is increased in CD4^+^ T cells isolated from the spinal cord of mice at the peak of EAE [33].

In EAE mice, increased GLUT1 expression and enhanced glycolysis in CD4⁺ T cells promote the activation of transforming growth factor-β (TGF-β). Under inflammatory conditions, when IL-6 levels are elevated in the spinal cord, the combined presence of TGF-β and IL-6 drives the differentiation of these T cells into proinflammatory Th17 cells [34, 35]. Increased mTORC1 signaling, HIF-1α expression, and glycolytic pathway activity play important roles in Th1 and Th17 induction while inhibiting Foxp3 expression and Treg differentiation. These alterations in T cell subsets ultimately enhance inflammatory responses in the CNS of EAE mice [32].

Th17 cells exhibit significant plasticity in response to environmental cues because of alterations in their metabolic pathways and functional profiles. In this context, increased glycolytic activity contributes to the induction of pathogenic Th17 cells in MS. In particular, the expression of the glycolytic enzyme glucose phosphate isomerase, which catalyzes the conversion of G6P to fructose-6-phosphate to support energy production and inflammatory cytokine release, is significantly greater in pathogenic Th17 cells isolated from the spinal cord of EAE mice than in steady-state Th17 cells from the intestine [36]. Several studies have shown that normalizing alterations in the glycolytic pathway in T cells improves symptoms in EAE mice. The inhibition of the expression of GAPDH, a key enzyme in the glycolytic pathway, in T cells isolated from the spleens of EAE mice using 3-bromopyruvate (3-BrPA) resulted in decreased IFN-γ production. Moreover, the administration of this inhibitor to EAE mice significantly attenuated encephalomyelitis [33]. The use of 2-deoxyglucose (2DG), which inhibits HK, and 6-phosphofructo-2-kinase/fructose-2,6-bisphosphatase 3 (PFKFB3), which are key enzymes in the glycolytic pathway, also ameliorates EAE [37]. Treated mice showed an increase in Treg cells, suppression of Th17 induction, and reduced immune cell infiltration into the CNS, ultimately preventing lesion progression [32, 37]. The inhibition of HIF-1α, a major inducer of glycolytic enzymes, by metformin in EAE mice also suppressed Th17 induction, limited T cell infiltration into the CNS, and reduced MS-related symptoms in mice [38]. Some molecules and drugs that target metabolic pathways and their role in lymphocyte differentiation and autoimmune disease progression are summarized in Table 1.

**Table 1:** Targeted metabolic pathways in Ms

MOLECULES AND DRUGS	Target metabolic pathway	Effects	References
3-BrPA	HK2 glycolysis (↓)	IFN-γ induction in T cells isolated from the spleen of EAE mice (↓). Encephalomyelitis (↓).	[33]
PFK158	PFKFB3 glycolysis (↓)	Th17 differentiation (↓). Disease activity (↓).	[37]
2DG	HK Glycolysis (↓).	Anti-inflammatory phenotype (↑). Disease progression in EAE (↓). CD69 MFI on CD8^+^ T cells from MS patient (↓).	[42, 72]
METFORMIN	HIF-1a Glycolysis (↓).	Th17 induction in EAE (↓). T cell infiltration into the CNS of EAE (↓). MS symptoms in EAE mice (↓).	[38]
GLATIRAMER	Glycolysis (↓).	ECAR and glycolytic enzyme levels in CD4^+^ cells of RRMS patients (↓). Improve disease.	[41]
DHODH	OXPHOS (↓). Glycolysis (↓).	T cell proliferation (↓).	[48]
IFN-Β	Glycolysis (↓).	ECAR and glycolytic enzyme levels in T cells of MS patients (↓). Improve disease.	[46]
SATURATED FATTY ACID SIDE CHAIN PHOSPHOLIPIDS	Bcl2 Mitochondrial dysfunction (↓).	Mitochondrial apoptosis-dependent pathways in T cells from EAE mice (↑). Autoreactive T cell apoptosis (↑). Experimental autoimmune encephalomyelitis (↓).	[56]
CATALASE	mROS Mitochondrial dysfunction (↓).	Treg cell death (↓). Th1 and Th17 (↓). Self-tolerance (↑).	[59]
OLIGOMYCIN	Mitochondrial ATP synthetizes Mitochondrial dysfunction (↓).	Th17 in a mouse model of MS (↓).	[60]
LINEZOLID	mEFG1 Mitochondrial dysfunction (↓).	Th1 and Th17 promotion (↓). Inflammatory cytokine production (↓).	[61]
ACC1 INHIBITOR	ACC1 FAS (↓).	Th17 differentiation (↓). Protects mice from EAE.	[18]
EI-03	FABP FAO (↑).	Tregs induction (↑). Th17 differentiation (↓). Alleviating symptoms in EAE mice.	[67]
ATORVASTATINE	HMG-COA reductase Cholesterol synthesis (↓).	Leading to Th1 to Th2 shift. MS progression in EAE mice (↓).	[68]
BTK INHIBITORS	BTK OXPHOS (↓).	OCR in B cells from MS patients (↓). B cells antigen-presenting function (↓).	[79]

*HK2* Hexokinase 2, *PFKFB3* 6-Phosphofructo-2-kinase/fructose-2,6-bisphosphatase 3, *2DG* 2-deoxy-D-glucose, *HK* Hexokinase, *HIF-1a* Hypoxia-inducible factor 1-alpha, *ECAR* Extracellular acidification rate, *RRMS* Relapsing-remitting multiple sclerosis, *DHODH* Dihydroorotate dehydrogenase, *OXPHOS* Oxidative phosphorylation, *mROS* Mitochondrial reactive oxygen species, *MEFG1* Mitochondrial elongation factor G1, *ACC1* Acetyl-CoA carboxylase 1, *FAS* Fatty acid synthesis, *FABP* Fatty acid binding protein, *FAO* Fatty acid oxidation, *BTK* Bruton’s tyrosine kinase, *OCR* Oxygen consumption rate

In addition to the direct modulation of glycolytic enzymes, other regulatory molecules play critical roles in controlling T cell metabolism and function in MS. For instance, nuclear receptor 77 (Nur77), a nuclear receptor involved in T cell activation, limits both mitochondrial respiration and glycolysis in T cells. In contrast, the phosphoinositide 3-kinase class III isoform (Pik3c3) is associated with an increased extracellular acidification rate (ECAR) following the differentiation of Th1 and Th17 subtypes. CD4^+^ T cells with Pik3c3 deficiency and Nur77 deletion exhibit impaired and enhanced glycolysis, respectively. Therefore, deficiencies in Pik3c3 and Nur77 have opposite effects on EAE progression. The contrasting roles of Nur77 and Pik3c3 highlight the critical importance of metabolic regulation in T cell function and EAE progression [33, 39, 40].

Alterations in the glycolytic pathway have also been reported in CD4^+^ T cells isolated from MS patients. However, there is still no consensus regarding the exact role of this pathway, and further research is needed. Evidence suggests that CD4^+^ T cells in MS patients predominantly rely on glycolysis to generate ATP. CD4^+^ T cells isolated from MS patients exhibit increased expression of GLUT1 and elevated levels of key glycolytic enzymes such as HK and phosphofructokinase [41]. Among CD4^+^ T cells from MS patients, the ECAR is also significantly increased [42, 43]. Compared with those in healthy controls, the levels of plasma lactate, a potential marker of glycolytic activity, were also found to be elevated in relapsing-remitting MS (RRMS) patients [44]. Although several studies have shown that compared with T cells from healthy individuals, CD4^+^ T cells from patients with RRMS display increased activity of both OXPHOS and glycolytic pathways, a greater proportion of ATP is generated via glycolysis than through the Krebs cycle. This reflects a metabolic shift favoring glycolytic energy production in MS-associated CD4⁺ T cells [45].

In contrast to the studies mentioned above, several other studies have shown impaired glycolytic pathways in CD4^+^ T cells from MS patients. CD4^+^ T cells from untreated MS patients exhibited statistically significant decreases in the expression of key glycolytic enzymes, including aldolase, enolase 1, HK1, and dihydrolipoamide S-acetyltransferase (DLAT). Additionally, the expression of GLUT1 tended to decrease. Disturbed metabolic pathways in CD4^+^ T cells are positively correlated with dysfunctional immune responses and may exacerbate the pathophysiology of MS [46]. Another study highlighted the important role of glycolysis in the differentiation of Tregs (iTregs) from conventional CD4^+^ T cells and its role in MS progression. CD4^+^ T cells from MS patients exhibit defects in the glycolytic pathway, resulting in reduced levels of enolase 1 and subsequently impaired iTreg induction, leading to worsening MS symptoms. Enolase 1 plays a key role by translocating to the nucleus to stimulate the expression of FOXP3, the key transcription factor involved in iTreg development [47].

Interestingly, standard MS treatments, such as glatiramer acetate, interferon-β (IFN-β), and dihydroorotate dehydrogenase (DHODH), significantly interfere with immune cell metabolic pathways. The ECAR and glycolytic enzyme levels in CD4^+^ T cells from patients with RRMS decreased after 12 months of glatiramer therapy and were similar to those in healthy controls [41]. DHODH also decreased OXPHOS activity and glycolysis in T cells and their proliferation [48]. Patients treated with IFN-β exhibited a similar reduction in the ECAR as healthy control CD4^+^ T cells did [46].

Taken together, these findings suggest that the correlation between the dysregulation of the glycolytic pathway in CD4^+^ T cells and MS pathogenesis is complex and multifaceted. While some studies have shown that reduced glycolytic activity in CD4^+^ T cells is correlated with defective iTreg differentiation, most studies, especially those in EAE models, suggest increased glycolysis, which promotes CD4^+^ T cell activation and the induction of proinflammatory Th1 and Th17 subsets. These differences emphasize the necessity of further studying the metabolic landscape of CD4 + T cells in patients at different stages of MS. It is also concluded that glycolytic pathways could be potential therapeutic targets for MS.

#### Mitochondrial dysfunction of CD4^+^ T cells in MS

Mitochondria are essential organelles in cells that perform critical functions. They play a critical role in cell metabolism through ATP production via the OXPHOS pathway and lipid metabolism. Additionally, mitochondria are involved in regulating apoptosis, and the production of reactive oxygen species (ROS) influences cell signaling pathways [49, 50]. By controlling these pathways, mitochondria can alter the function and fate of immune cells [51]. Several mitochondrial defects have also been observed in T cells from MS patients and an EAE mouse model [52, 53]. Mitochondrial defects in T cells disrupt cellular metabolism, apoptosis, and signaling pathways, ultimately exacerbating disease.

Activated CD4^+^ memory T cells in patients with MS have increased CCR5 expression, which enhances their migration into the CNS is associated with reduced susceptibility to apoptosis because of decreased mitochondrial transmembrane potential. This defect in mitochondrial-mediated apoptotic elimination leads to the prolonged survival of autoreactive memory CD4^+^ T cells in circulation and limits their elimination in the CNS, contributing to MS progression [54, 55]. Saturated fatty acid side chain phospholipids prevent Bcl-2 from interacting with the pro-apoptotic proteins Bim and Bad and reduce their phosphorylation, which leads to the normalization of mitochondrial apoptosis-dependent pathways. This process can ameliorate EAE by inducing autoreactive T cell apoptosis [56]. Defects in mitochondria in CD4^+^ T cells from MS patients also result in reduced superoxide dismutase (SOD) and glutathione peroxidase (GPX) production, exacerbating oxidative stress and inducing HSP70 production. Toxic aggregation and carbonylated protein accumulation in CD4^+^ T cells increase their vulnerability to oxidative damage and may contribute to MS progression [45]. Notably, increased levels of ROS activate the latent form of TGF-β in inflammatory CNS CD4^+^ T cells in MS, which shifts FOXP3 expression to RORγT expression, ultimately leading to Th17 induction and EAE progression [35]. Furthermore, the morphology and function of Tregs from MS patients are altered. Defects are indicated by a longer mitochondrial ellipsoid length, lower sphericity, and a lower maximum respiratory rate in affected individuals than in healthy individuals [57, 58]. Mitochondrial oxidative stress also occurs in FOXP3^+^ Tregs in MS. Increased ROS in these cells leads to impaired lysosomal function and promotes the DNA damage response (DDR), followed by cell death. Capturing mtROS either chemically or by targeted expression of the antioxidant enzyme catalase restores Treg survival and attenuates Th1 and Th17 responses, promoting self-tolerance [59]. Mitochondrial dysfunction and alterations in metabolic and signaling pathways in the T cells of patients with MS lead to the differentiation of inflammatory T cell subsets and the induction of inflammatory cytokines through transcriptional, translational, and epigenetic mechanisms. As a consequence of mitochondrial dysfunction, elevated OXPHOS in MS T cells further drives proinflammatory responses by modulating key signaling pathways. Increased OXPHOS in MS T cells activates key transcription factors, such as basic leucine zipper ATF-like transcription factor (BATF) and signal transducer and activator of transcription 3 (STAT3), which regulate mTORC1 and HIF-1α pathways and epigenetic modifications. Collectively, these processes increase Th17 differentiation, exacerbating the disease. Notably, inhibition of mitochondrial ATP has been shown to suppress Th17 pathogenicity in a mouse model of MS by blocking the expression of Th17-specific pathogenic gene signatures [60].

In addition to signaling and transcriptional regulation, mitochondrial dysfunction in MS T cells also involves the control of protein translation, further amplifying inflammatory responses. One example is the upregulation of mEF-G1, a translation factor that leads to ETC synthesis, which is upregulated in MS T cells, followed by ETC activation and increased NAD^+^ production, which is necessary for inflammatory cytokine production. Blocking mEF-G1 with Linezolid disrupts the integrity of the ETC and the NAD^+^/NADH ratio, inhibiting Th1 and Th17 promotion and inflammatory cytokine production [61].

In summary, mitochondrial dysfunction in T cells contributes to MS pathophysiology by impairing apoptosis, increasing oxidative stress, and promoting inflammatory Th17 responses. Defective mitochondria disrupt metabolic and signaling pathways, sustaining autoreactive T cells and weakening immune regulation. Targeting mitochondrial pathways, such as reducing ROS or inhibiting OXPHOS, could restore immune balance and slow MS progression, highlighting mitochondria as a key therapeutic focus.

#### Dysregulated lipid metabolism in CD4^+^ T cells in MS

Lipids are essential components of all cells and organelle membranes. They provide structural support, act as energy sources, and function as signaling platforms. In patients with MS, changes in the lipid profile are observed not only in the bloodstream but also in T lymphocytes. Several studies have reported alterations in intracellular pathways involved in lipid metabolism in T cells from patients with MS, highlighting the important role of lipid dynamics in the MS process. While de novo fatty acid synthesis is essential for Th17 differentiation, Tregs depend more on exogenous fatty acids. Specific deletion of ACC1 in T cells results in decreased Th17 differentiation and protects mice from EAE [18, 62]. Lipid mass spectrometry revealed that compared with those from healthy controls, the lipid composition of CD4⁺ T cells from MS patients is altered, leading to changes in membrane properties and signaling that promote abnormal adhesion and T cell infiltration through the blood–brain barrier (BBB) into the CNS. Furthermore, an increase in the expression of cardiolipin, the main lipid component of the mitochondrial membrane, indicated that these cells were in a state of mitochondrial stress in MS [63]. Alterations in signaling and metabolic pathways in CD4^+^ T cells also follow changes in blood lipid profiles in MS patients, contributing to disease progression. Oleic acid induces FAO and OXPHOS metabolic pathways, stimulates Foxp3 expression, and increases p-STAT5 levels, all of which are required for the suppressive function of Tregs, whereas elevated arachidonic acid levels lead to proinflammatory T cell responses. In MS, elevated arachidonic acid and decreased oleic acid levels impair Treg cell function and, conversely, increase the inflammatory environment. These effects can be restored by the supply of oleic acid [64]. The levels of propionate, a short-chain fatty acid (SCFA), are reduced in the serum and stool of MS patients. Supplementation with propionate restored T cell mitochondrial function and morphology. It also enhances mTORC1 activity and glucose oxidation in T cell metabolic pathways. These changes increase IL-10 production and suppress Th17 differentiation and IL-17 production, partly through the histone deacetylase inhibitory activity of propionate-derived acetyl-CoA. Additionally, propionate activates the p38 MAPK signaling pathway, which promotes p-STAT3 activation and further enhances IL-10 production in Tregs, supporting immune regulation [57, 65, 66].

Dysregulation of epidermal fatty acid-binding protein (E-FABP), an intracellular lipid chaperone, is also observed in T lymphocytes from MS patients. EI-03, a synthetic small-molecule inhibitor, selectively binds to the lipid binding pocket of E-FABP, thereby inhibiting its activity. The inhibition of E-FABP activity by EI-03 leads to the induction of peroxisome proliferator-activated receptor gamma **(**PPAR-γ), ultimately increasing the number of Tregs and decreasing the number of Th17 cells, thereby alleviating symptoms in EAE mice [67]. Another change in T cell lipid metabolism in EAE is an increase in the activity of the cholesterol synthesis pathway. This metabolic change leads to an increase in phosphorylated STAT4 and subsequent Th1 induction. The inhibition of HMG-CoA reductase, the main enzyme of the cholesterol synthesis pathway, by atorvastatin in T cells decreases phosphorylated STAT4 levels, while phosphorylated STAT6 levels increase, ultimately leading to a Th1-to-Th2 shift and ameliorating MS progression in EAE mice [68].

Taken together, these data highlight the alterations in lipid profiles and lipid metabolism that play critical roles in T cell function and differentiation in the context of an inflammatory milieu that exacerbates disease progression. Therefore, the modulation of lipid metabolism is a promising therapeutic strategy for MS.

### Dysregulation of metabolic pathways and mitochondrial dysfunction in CD8^+^ T cell metabolism in MS

CD8⁺ T cells are increased in the CNS of individuals with MS, where myelin-specific cells drive damage via perforin, granzyme, and IFN-γ [69]. Metabolic alterations in these cells have also been reported in MS patients and EAE models (Fig. 4B). Activation of myelin-specific CD8⁺ T cells in the CNS induces a shift toward glycolysis, promoting effector differentiation and increased cytotoxic activity that drives myelin damage [70, 71]. The inhibition of glycolysis with 2DG reduces CD69 expression in CD8⁺ T cells in MS, underscoring its role in their activation and disease pathogenesis. CD8⁺ T cell subsets in MS patients had higher mitochondrial mass and higher mitochondrial membrane potential than those in healthy controls did [72].

Dimethyl fumarate (DMF) reprograms metabolic activity in human CD4⁺ and CD8⁺ T cells by depleting glutathione (GSH), impairing antioxidant capacity and inducing caspase-mediated apoptosis. This leads to increased mitochondrial ROS, membrane potential, and cytochrome c release, along with reduced mitochondrial oxygen consumption rate (OCR) [73].

Together, these findings indicate that CD8^+^ T cells, particularly short-lived effector cells, contribute to CNS myelin damage through their cytotoxic activities and metabolic alterations. Increased glycolysis and mitochondrial dysfunction in these cells highlight the potential of metabolic pathways as therapeutic targets for MS.

### Dysregulation of B cell metabolism in MS

The role of B cells in MS pathogenesis has recently garnered considerable interest, particularly since B cell depletion therapies targeting CD20 have been shown to slow disease progression [74]. In MS, B cells with elevated MHC class II, CD40, and CD80 expression function as potent APCs that present self-antigens to T cells, leading to increased T cell activity and myelin damage [75, 76]. Additionally, B cells contribute to MS development through the secretion of autoantibodies and various cytokines [77]. B cells from MS patients produce elevated levels of IL-6, a cytokine crucial for the differentiation of encephalitogenic Th17 cells and subsequent myelin damage [78]. While metabolic changes in T cells have been extensively studied, research has also highlighted alterations in B cell metabolic pathways and their potential role in MS pathogenesis (Fig. 4C).

#### Dysregulated glycolytic pathway in B cells in MS

Emerging evidence suggests that B cells from patients with MS undergo significant metabolic reprogramming. However, direct evidence assessing the glycolytic pathway specifically in B cells from MS patients remains limited. Pharmacological inhibition of Bruton’s tyrosine kinase (BTK) can normalize the disturbed metabolic profile of B cells from MS patients, leading to decreased antigen-presenting capacity and reduced B cell–T cell interactions. Importantly, these effects are due mainly to the recovery of mitochondrial respiration rather than changes in glycolysis [79]. In contrast, metabolomic analyses in relapsing–remitting MS have revealed elevated levels of glycolytic intermediates, indicating a systemic increase in glycolytic flux in circulating immune cells, possibly including B cells [42]. Furthermore, intrathecal B cells in the CSF and MS lesions produce autoantibodies that target glycolytic enzymes, such as triosephosphate isomerase (TPI) and GAPDH. Although these findings do not directly prove that glycolytic dysregulation occurs within B cells, they suggest that an immune response is directed toward components of the glycolytic pathway [80]. Collectively, these findings underscore the need for more precise investigations to elucidate the role of glycolytic dysregulation in B cell pathophysiology in MS, as such insights may reveal novel metabolic targets for therapeutic intervention.

#### Dysregulated OXPHOS pathway in B cells in MS

In addition to glycolysis, OXPHOS is also elevated in the B cells of patients with MS. Given these metabolic alterations, targeting key signaling molecules in B cells is a promising therapeutic approach. BTK, a critical regulator of B cell activation and signaling, is a key target in this context. BTK inhibition not only reduces the OCR in these cells but also attenuates their antigen-presenting function [79]. Additionally, BTK inhibition in EAE mice reduces OXPHOS in B cells, leading to the release of anti-inflammatory cytokines, which ultimately contributes to decreased neuroinflammation [81]. These findings suggest that metabolic alterations in B cells contribute to MS pathogenesis and may represent potential therapeutic targets.

### Integrated metabolic signature of lymphocytes in MS

Mounting evidence indicates that metabolic reprogramming is a central mechanism driving lymphocyte pathogenicity in MS rather than merely a consequence of inflammation [45]. Across studies in MS patients and EAE models, CD4⁺ T cells consistently exhibit a shift toward glycolysis driven by key regulators such as mTORC1, HIF-1α, and c-Myc [32]. This metabolic state promotes the differentiation of proinflammatory Th1 and Th17 cells while impairing Treg development, thereby disrupting immune tolerance [32]. In parallel, mitochondrial dysfunction further amplifies disease progression by increasing ROS production, enhancing inflammatory signaling, and allowing autoreactive T cells to escape apoptosis [52, 53]. Alterations in lipid metabolism provide an additional layer of metabolic control over immune responses in MS. Increased fatty acid synthesis favors inflammatory T cell differentiation, whereas reduced availability of regulatory lipid metabolites impairs Treg function [18, 62]. However, notably, such metabolic disorders are not limited to T cells alone. B cells have also been reported to show enhanced OXPHOS and metabolic function, leading to an improvement in antigen presentation efficiency and stimulation of autoreactive T cells [76, 79]. Such studies have shown that metabolic disorders affect various types of lymphocytes and can facilitate ongoing autoimmune processes. Therefore, alterations in glucose metabolism, mitochondrial function, and lipid metabolism contribute to an inflammatory response by lymphocytes at the expense of the inhibition of regulatory pathways.

## Rheumatoid arthritis

**Fig. 5:**
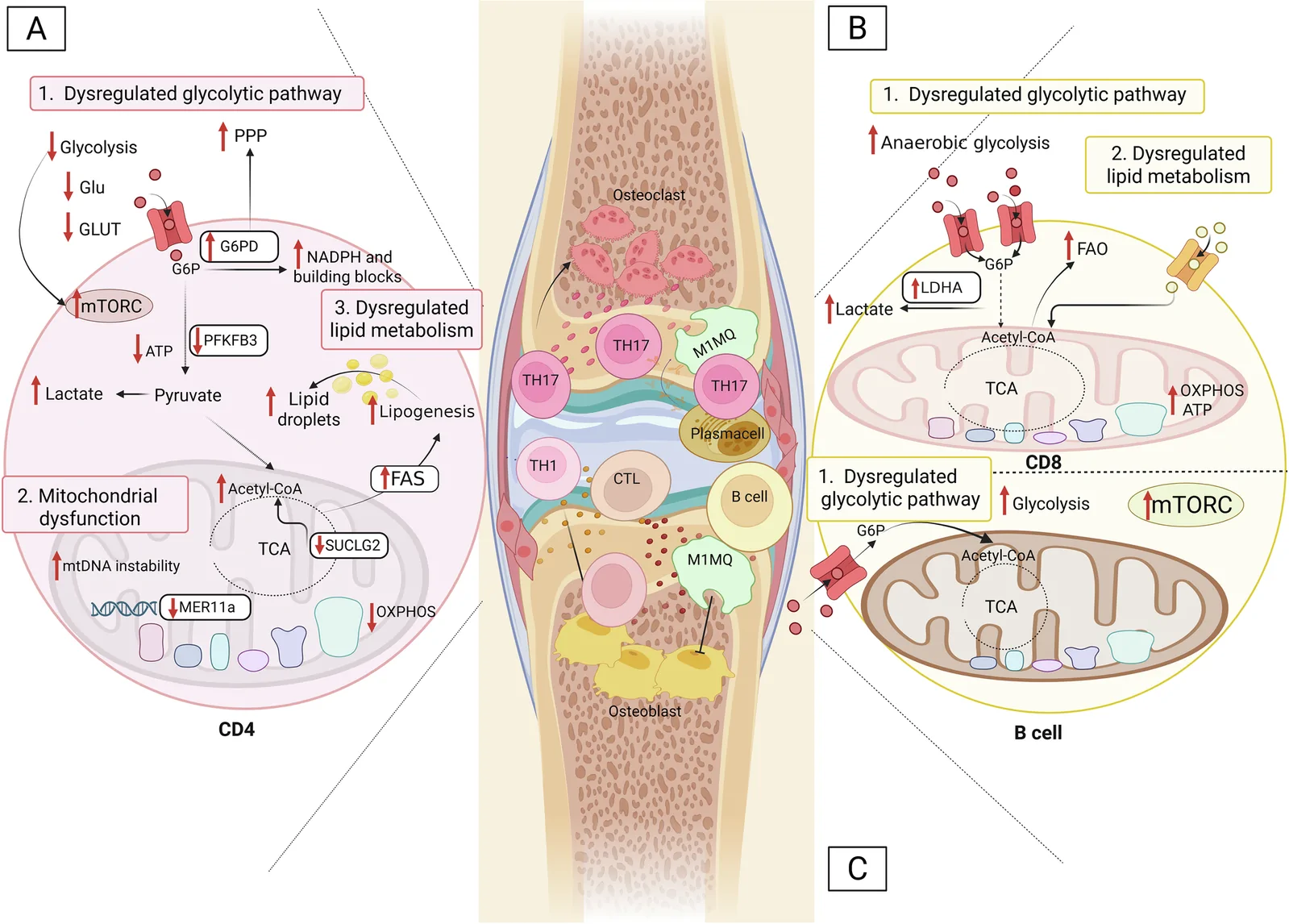
Metabolic alterations in T cells and B cells in rheumatoid arthritis. **A** CD4^+^ T cells in RA exhibit impaired glycolysis, with glucose shunted toward the pentose phosphate pathway, leading to reduced stress and hyperproliferation. Additionally, defects in mitochondrial enzymes, including SUCLG2 and MRE11a, have distinct consequences. SUCLG2 deficiency leads to acetyl-CoA accumulation, subsequently increasing tubulin acetylation and tissue invasion of T cells. Furthermore, mitochondrial DNA instability in CD4^+^ T cells due to MRE11a deficiency results in inflammasome activation. Dysregulated lipid metabolism in these cells, including de novo fatty acid synthesis and lipogenesis, also contributes to podosome formation and the invasiveness of CD4^+^ T cells. **B** CD8^+^ T cells rely on glycolysis and FAO to meet their ATP demands for the effector function of autoreactive CD8^+^ T cells. **C** B cells in RA display increased glycolysis, enhancing their capacity to produce autoantibodies. The figure was created with BioRender software

RA is classified as both a T cell-mediated and a systemic autoimmune disorder, marked by persistent inflammation in the joints, synovial hyperplasia, and the degradation of bone and cartilage. Substantial evidence supports the notion that the recognition of autoantigens by specific T cells plays a vital role in the pathophysiological mechanisms underlying RA [82]. While immune dysregulation is a well-established driver of RA pathogenesis, emerging evidence highlights that metabolic changes in immune cells contribute to RA pathogenesis by shaping their effector functions (Fig. 5).

### Dysregulation of CD4^+^ T cell metabolism in RA

In RA, CD4^+^ T cells are the predominant lymphocytes in synovial tissue and play a critical role in inflammatory processes within the synovial membrane. These cells contribute to autoantibody production by PCs, produce inflammatory cytokines, and activate macrophages and synoviocytes [83]. Additionally, RA CD4^+^ T cells facilitate bone erosion through osteoclast activation and inhibit collagen production by synoviocytes [84]. Alterations in metabolic pathways represent a fundamental determinant of T cell function and differentiation under both normal and disease conditions. While the relationship between T cell metabolic reprogramming and the pathogenesis of RA has not yet been fully elucidated, several pivotal metabolic changes have been characterized (Fig. 5A).

#### Dysregulated glycolytic pathway in CD4^+^ T cells in RA

Seminal work from Weyand and colleagues first demonstrated that the activation of the glycolytic regulator PFKFB3 is not appropriate for naïve CD4^+^ T cells from patients with RA. As a consequence, glucose metabolism is diverted away from glycolysis toward the PPP, resulting in reduced ATP production despite increased glucose utilization. This metabolic rewiring distinguishes RA T cells from activated healthy T cells and contributes to disease pathogenesis [85, 86]. Building on these observations, Weyand and coworkers further demonstrated that reduced PFKFB3 activity redirects glucose into the PPP through increased G6PD activity, thereby generating excessive NADPH and creating a state of reductive stress. Elevated NADPH levels contribute to the consumption of ROS, inducing a state of reductive stress that hampers the activation of the cell cycle kinase ataxia telangiectasia mutated (ATM). This impairment allows RA T cells to bypass the G2/M cell cycle checkpoint, leading to hyperproliferation. Additionally, ATM deficiency skews naïve CD4⁺ T cell differentiation toward Th1 and Th17 lineages, creating a pool of inflammation-prone T cells. Furthermore, inducing elevated ROS levels through menadione (a 1,4-naphthoquinone analog) or buthionine sulfoximine (BSO) suppresses proinflammatory transcription factors and cytokines, thereby mitigating T cell hyperproliferation and synovial inflammation [87]. Importantly, targeting glucose metabolism with 3-BrPA, an inhibitor of HK2, markedly reduced arthritis and disease symptoms in a mouse model of arthritis. This treatment was associated with a notable increase in Tregs and a decrease in Th17 cells [88].

Another metabolic feature associated with naïve RA CD4^+^ T cells is mTOR signaling, which plays a central role in T cell development and differentiation. In RA, mislocalization of the energy sensor AMPK fails to occur in low ATP states, allowing mTORC1 to drive anabolic processes in RA T cells. Consequently, sustained activation of mTORC1 further commits RA T cells to Th1 and Th17 differentiation [89].

Additionally, in RA patients, circulating Tfh (cTfh) cells exhibit increased B cell helper activity, likely resulting from enhanced glycolysis mediated by the CXCL13–CXCR5 axis. In contrast, peripheral helper T (Tph) cells show heightened cytotoxic potential, which may be associated with increased mitochondrial ROS production driven by the CCL2–CCR2 axis [90].

Furthermore, epigallocatechin-3-gallate (EGCG), a polyphenol found in green tea, was shown to mitigate arthritis and prevent joint degradation in a mouse model of arthritis. This protective effect is achieved through the inhibition of mTOR/HIF-1α signaling and the expression of glycolytic factors, such as Glut-1 and LDH-A, which promote Th17 cell differentiation [91].

Importantly, in the hypoxic and chronically inflamed synovial microenvironment of patients with RA, intensified glycolytic activity in synovial fibroblasts and macrophages leads to excessive lactate accumulation. This lactate buildup stimulates the expression of the lactate transporter solute carrier family 5 member 12 (SLC5A12) in CD4⁺ T cells. SLC5A12-mediated lactate uptake induces IL-17 production via nuclear PKM2/STAT3 signaling and increases fatty acid synthesis while also restricting synovial T cell motility, leading to T cell retention in inflamed areas [92].

In summary, metabolic reprogramming in RA T cells enhances their invasion into the synovial microenvironment while simultaneously inhibiting their motility. This dual effect leads to T cell retention within the inflamed synovium.

#### Mitochondrial dysfunction and dysregulated fatty acid metabolism in CD4^+^ T cells in RA

RA CD4⁺ T cells also exhibit defects in mitochondrial oxygen consumption and ATP production. Specifically, OXPHOS is compromised because of a deficiency in the GDP-forming β-subunit of succinate-CoA ligase (SUCLG2), a mitochondrial enzyme that catalyzes the conversion of succinyl-CoA into succinate and acetyl-CoA. This deficiency leads to the accumulation of acetyl-CoA, resulting in tubulin hyperacetylation and the generation of hyperinflammatory, tissue-invasive T cells [93]. In RA CD4⁺ T cells, the metabolic state is characterized by high NADPH and acetyl-CoA levels, along with the upregulation of enzymes involved in de novo fatty acid synthesis, such as FASN and acetyl-CoA carboxylase (ACACA). This metabolic phenotype facilitates lipogenesis and the accumulation of lipid droplets in RA T cells. Additionally, this metabolic shift increases the locomotion program through the expression of the podosome scaffolding protein TKS5, enabling T cells to form invasive membrane structures [94]. In addition to the metabolic alteration in fatty acid metabolism, uncoupling protein 2 (UCP2) serves as a critical regulator connecting the glycolysis and FAO pathways. In RA, Tregs exhibit elevated UCP2 expression, which disrupts metabolic equilibrium by suppressing the expression of carnitine palmitoyl transferase 2 (CPT2), the rate-limiting enzyme of the FAO pathway. The resulting impairment of FAO activates caveolae-mediated endocytosis, leading to decreased surface expression of CTLA4 and reduced Treg suppressive function. Consequently, the dysfunction of Tregs contributes to sustained inflammatory responses within the synovium [95].

Furthermore, mitochondrial fitness and ATP production are compromised because of the deficiency of the DNA repair nuclease MRE11 meiotic recombination 11 homolog A (MRE11a). Insufficient MRE11a activity leads to mitochondrial DNA instability, mtDNA leakage into the cytoplasm, and subsequent inflammasome activation [96].

Mitochondria control ER biogenesis by supplying key metabolites and maintaining redox balance. In T cells with healthy mitochondria, the TCA cycle supports aspartate production and NAD⁺ regeneration; these processes, in turn, keep the ER chaperone BiP/GRP78 cycling properly and limit unnecessary ER expansion. In RA, synovial T cells have a mitochondrial defect that reduces their ability to produce mitochondrial aspartate. This shortage disrupts the ER balance by interfering with the regulation of NAD⁺ and BiP/GRP78. As a result, the ER becomes enlarged with more ribosomes, which increases the production of transmembrane TNF-α during protein synthesis. These ER-enriched T cells are the main source of TNF-α in RA joints. Experiments have shown that restoring mitochondrial function or supplying aspartate reduces ER size, decreases TNF-α levels, and relieves joint inflammation in RA models. These findings indicate that a mitochondrial–ER metabolic interaction directly contributes to the TNF-α-driven disease process in RA [97].

Consequently, defects in mitochondrial function and alterations in metabolic enzymes drive the proliferation and inflammatory characteristics of RA CD4⁺ T cells. Additionally, metabolic dysregulation can lead to impaired RA Treg function, exacerbating inflammatory conditions.

### Dysregulation of CD8^+^ T cell metabolism in RA

CD8⁺ T cells, including tissue-resident memory CD8⁺ T cells, are elevated in the synovium of patients with RA and contribute to joint destruction through the secretion of inflammatory mediators in response to antigens. Cytotoxic CD8⁺ T cells specifically target citrullinated antigens, leading to joint tissue damage in anti-citrullinated protein antibody-positive (ACPA⁺) RA [98]. Additionally, CD8⁺ T cells play a crucial role in the formation of ectopic GCs in the RA synovium [99].

#### Dysregulated glycolytic and fatty acid pathways in CD8^+^ T cells in RA

Research has established a link between the metabolic reprogramming of CD8⁺ T cells and the pathogenesis of RA. CD8⁺ T cells from both peripheral blood and the synovial membrane exhibit increased expression of glycolytic enzymes, such as LDHA, and meet their metabolic needs primarily through aerobic glycolysis. LDHA is essential for converting pyruvate to lactate, which supports the proinflammatory and cytolytic functions of CD8⁺ T cells. Furthermore, peripheral CD8⁺ T cells in RA can survive in hypoxic and low-glucose environments, such as those in the synovial membrane, by relying on glycolysis and glutaminolysis for energy production [100].

Metabolic alterations in RA CD8^+^ T cells occur not only in the glycolytic pathway but also in fatty acid metabolism. Fatty acid transporters, including FABP4 and G protein–coupled receptor 84 (GPR84), are upregulated in effector and memory CD8⁺ T cells from RA patients compared with those from healthy controls. Blocking fatty acid metabolism in vitro significantly diminishes the effector functions of CD8⁺ T cells [101].

In conclusion, CD8⁺ T cells, including tissue-resident memory CD8⁺ T cells, play a significant role in joint destruction in RA through their proinflammatory activities and metabolic reprogramming. Their reliance on glycolysis and the upregulation of fatty acid transporters highlight potential therapeutic targets to mitigate inflammation and joint damage in patients with RA (Fig. 5B).

### Dysregulation of B cell metabolism in RA

B cells in RA produce rheumatoid factors and anti-citrullinated protein antibodies, driving immune complex formation and complement activation in joints [102]. They also act as APCs to activate autoreactive T cells [103], and support tertiary lymphoid tissues through lymphotoxin α and β, which are correlated with disease severity [104]. However, B cell metabolism in RA remains less well understood (Fig. 5C).

Compared with those in healthy controls, peripheral CD19⁺ B cells in patients with RA exhibit increased glycolysis, which contributes to B cell hyperactivity. Elevated serum IL-27 levels in patients with RA correlate positively with disease activity, plasma cell frequency, and autoantibody levels [105]. IL-27 enhances glycolysis in RA B cells by activating mTOR signaling. Moreover, PD-1⁺ B cells accumulate in the RA synovium and rely heavily on glycolysis. Under hypoxic conditions that mimic the joint microenvironment, compared with their PD-1⁻ counterparts, RA PD-1⁺ B cells express higher levels of CXCR3, CD80, CD86, IL-1β, and GM-CSF [106]. Specifically, inhibition of glycolysis using 2DG markedly diminished joint inflammation, the activation of both adaptive and innate immune cells, and the synthesis of pathogenic autoantibodies [107]. Additionally, the administration of the specific hexokinase inhibitor lonidamine (LND) in a mouse model of arthritis resulted in a reduction in antibody production against IgG1, IgG2a, and IgG2b, thereby mitigating joint inflammation and damage. In a mouse model of RA, a mutation in the neutrophil cytosolic factor 1 (NCF1) gene led to a deficiency in ROS, impairing the ability to resist arthritis. These findings suggest that ROS play a role in mediating B cell tolerance to autoantigens [108].

B cell dysregulation in RA, marked by increased glycolysis and PD-1⁺ B cell activity, significantly contributes to disease progression through autoantibody production and inflammation. These findings suggest that targeting these metabolic changes may offer new therapeutic strategies for treating RA.

### Metabolic integration and functional consequences in RA

A landmark series of studies from the Weyand-Goronzy laboratory revealed that the metabolic program of RA differs fundamentally from that of many other autoimmune diseases. Unlike those with increased glycolysis, naïve RA CD4^ +^ T cells exhibit defective induction of PFKFB3, which diverts glucose into the PPP [85, 87, 109]. This metabolic rewiring increases NADPH production, decreases intracellular ROS levels, suppresses ATM activation, and promotes differentiation into pathogenic Th1 and Th17 cells [87, 110]. Furthermore, the abnormal function of mTORC1 and AMPK contributes to anabolic metabolism in inflammatory T cells [89].

Mitochondrial dysfunction represents another key feature of RA lymphocytes. Defects in mitochondrial enzymes and mitochondrial DNA maintenance impair OXPHOS and ATP generation while promoting the accumulation of biosynthetic intermediates such as acetyl-CoA. These alterations drive abnormal lipid synthesis, cytoskeletal remodeling, and increased invasiveness of synovial T cells [96]. Moreover, mitochondrial abnormalities disrupt Treg metabolism, reducing T cell suppressive capacity and further amplifying immune dysregulation [95]. In addition to CD4^+^ T cells, CD8⁺ T cells and B cells in RA exhibit enhanced glycolysis and altered lipid metabolism, supporting their survival and promoting autoantibody production and inflammatory cytokine release [101, 106].

Importantly, metabolic changes in RA lymphocytes are closely linked to the inflammatory microenvironment of the synovium. Metabolites such as lactate not only reflect metabolic activity but also actively promote inflammatory signaling, cytokine production, and the retention of immune cells within inflamed tissues [92]. These findings support a model in which coordinated alterations in glucose, mitochondrial, and lipid metabolism promote persistent immune activation while impairing regulatory mechanisms. Together, this integrated metabolic imbalance represents a central mechanism driving RA pathogenesis and highlights immunometabolism as a promising target for therapeutic intervention (Table 2) [107]. Notably, these metabolic features share important similarities with those observed in other autoimmune diseases, suggesting that common immunometabolic pathways are involved in chronic autoimmunity.

**Table 2:** Targeted metabolic pathways in RA

Molecules and drugs	Target metabolic pathway	Effects	References
3-BrPA	HK2 Glycolysis (↓).	Tregs in mouse model of arthritis (↑). Th17 in mouse model of arthritis (↓). Disease symptoms in the mouse model of arthritis (↓).	[88]
EGCG	mTOR/HIF-1α Glycolysis (↓).	Th17 promotion (↓). Joint degradation in a mouse model of arthritis (↓).	[91]
2DG	HK Glycolysis (↓).	Adaptive and innate immune cells (↓). Synthesis of pathogenic autoantibodies (↓). Joint inflammation (↓).	[107]
LND	HK Glycolysis (↓).	Antibody production IgG1, IgG2a, and IgG2b in mouse model of arthritis (↓). Joint inflammation (↓).	[192]

*HK2* Hexokinase 2, *mTOR* Mechanistic target of rapamycin, *HIF-1α* Hypoxia-inducible factor 1-alpha, *2DG* 2-deoxy-D-glucose, *HK* Hexokinase, *LND* Lonidamine, *IgG* Immunoglobulin G

## Type 1 diabetes

T1D is an autoimmune disorder mediated by T cells and characterized by persistent inflammation of the pancreatic islets of Langerhans. This inflammatory process typically involves the infiltration of both adaptive and innate immune cells, ultimately leading to the destruction of insulin-producing β-cells. Once a critical threshold of β-cell mass is compromised or destroyed, hyperglycemia occurs, resulting in the clinical onset of diabetes [111–113].

**Fig. 6:**
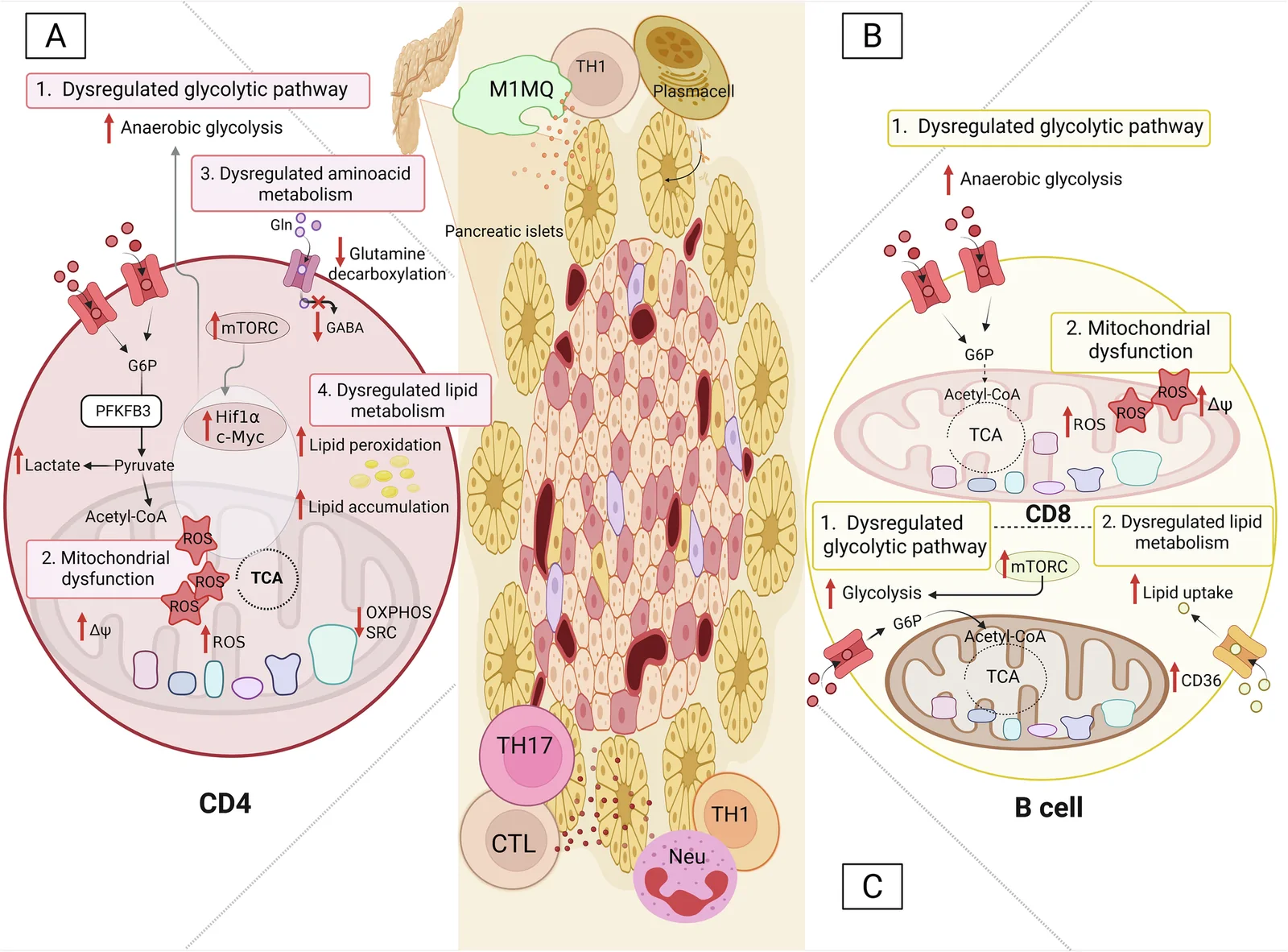
Metabolic alterations in T cells and B cells in type 1 diabetes. **A** In T1D, autoreactive CD4⁺ T cells exhibit a pronounced reliance on aerobic glycolysis to meet the metabolic demands associated with rapid clonal expansion and skewing toward proinflammatory Th1 and Th17 lineages, accompanied by a reduction in Treg induction. Mitochondrial abnormalities, including mitochondrial MHP, further promote TH1 differentiation and increase inflammatory cytokine production. Elevated ROS drive a metabolic shift toward glycolysis, resulting in increased accumulation of oxidized lipids and heightened autoreactive T cell activation. Defects in GABA production additionally indicate impaired Treg generation in T1D. Dysregulated lipid metabolism and increased lipid peroxidation contribute to mitochondrial dysfunction and apoptosis. **B** CD8⁺ T cells in T1D similarly upregulate glycolytic activity, while mitochondrial hyperpolarization augments their cytotoxic function. Elevated ROS levels further potentiate their effector responses. **C** B cells undergo a metabolic shift toward glycolysis, supporting the enhanced production of proinflammatory cytokines. Increased FA uptake in these cells also facilitates autoantibody generation. The figure was created with BioRender software

Our understanding of T1D pathogenesis is largely derived from studies on nonobese diabetic (NOD) mice, which have a genetic predisposition to autoimmune diabetes and share several features with humans with T1D [114]. Additionally, research on metabolic pathways in lymphocytes from NOD mice has provided insights that may be relevant to the progression of the disease in humans (Fig. 6).

### Dysregulation of CD4^+^ T cell metabolism in T1D

The disruption of insulin-secreting β-cells in T1D is mediated by innate and adaptive immune cells. Among the immune cells involved, autoreactive CD4⁺ T and CD8⁺ T cells are the most important cells that play a fundamental role in T1D pathogenesis [111]. The transfer or deletion of these cells can induce or prevent disease, respectively [115]. Among CD4⁺ T cells, Th1 and Th17 cells play interconnected roles in disease pathogenesis. In the early stages of T1D, Th1 cells increase and produce IFN-γ, which activates CD8⁺ T cells and triggers β-apoptosis [116, 117]. During advanced β-cell autoimmunity, increased Th17 cells induce neutrophil recruitment and amplify autoimmune responses through the production of IL-17 and IL-22 [118]. As mentioned above, changes in metabolic pathways are linked to the pathogenesis of autoimmunity through effects on T cell differentiation and function (Fig. 6A). The link between changes in T cell metabolism and their involvement in T1D development is described below.

#### Dysregulated glycolytic pathway in CD4^+^ T cells in T1D

In T1D, autoreactive CD4⁺ T cells prefer aerobic glycolysis over OXPHOS to meet their bioenergetic demands during activation. While compared with OXPHOS, glycolysis yields substantially less ATP per glucose molecule, its rapid ATP generation kinetics are critical for supporting T cell activation, rapid clonal expansion, and cytokine production, leading to sustained autoimmune responses to pancreatic β-cells [119]. This highlights the therapeutic potential of targeting this metabolic axis in T1D pathogenesis. Novel therapeutic strategies targeting glycolytic pathways in T1D converge on immune modulation through divergent mechanisms. The inhibition of mTORC1, a key signaling axis upstream of the glycolytic pathway, by rapamycin promotes the expansion of functional Tregs in human T1D cells in vitro. However, in patients with T1D, rapamycin combined with IL-2, despite increasing Tregs numbers, was associated with a transient impairment of β-cell function [120, 121]. The increase in glycolysis in the T cells of T1D patients is also driven by key upstream transcription factors, making their inhibition a promising therapeutic strategy. Furthermore, metformin, the first-line treatment for T1D, has been shown to target key upstream transcription factors of the glycolytic pathway, particularly HIF-1α and c-Myc, thereby suppressing the expression of glycolytic enzymes. This metabolic reprogramming attenuated glycolysis in T cells, leading to reduced Th1 and Th17 differentiation and enhanced Treg development in vitro.

NOD mice treated with metformin showed alleviated autoimmune insulitis and reduced numbers of Th1 and Th17 cells in the spleen [122, 123]. The pharmacological inhibitor PFK15, which targets the rate-limiting enzyme PFKFB3, promotes the functional exhaustion of autoreactive CD4⁺ T cells, as evidenced by persistent PD-1/LAG-3 expression and irreversible hyporesponsiveness to β-cell antigens in vitro. Additionally, in an adoptive transfer model, PFK15 treatment delayed the onset of T1D by inducing functional and metabolic exhaustion of diabetogenic CD4⁺ T cell clones [119]. These results demonstrate that autoreactive CD4⁺ T cells in T1D undergo a pathogenic metabolic shift to aerobic glycolysis at the expense of OXPHOS. Targeting this metabolic heterogeneity through the inhibition of glycolytic enzymes (PFKFB3 via PFK15), upstream signaling molecules (mTORC1 via rapamycin), and transcription factors (HIF-1α/c-Myc via metformin) impairs pathogenic T cell responses by inducing functional exhaustion in CD4⁺ T cells, enhancing Treg suppressive function, and suppressing Th1 and Th17 differentiation.

#### Mitochondrial dysfunction of CD4^+^ T cells in T1D

Mitochondrial defects are observed in the CD4⁺ T cells of T1D patients and NOD model mice. These defects play a crucial role in the pathogenesis of T1D, leading to metabolic disturbances and increased immune cell activation. The GTPase of immune-associated nucleotide-binding protein 5 (GIMAP5) is an important mitochondrial outer membrane protein that plays a vital role in preserving T cell longevity through its effects on mitochondrial calcium buffering and the stabilization of antiapoptotic proteins. Mutations in GIMAP5 have been shown to cause lymphopenia and disturbed immune regulation in T cells, leading to the development of autoimmune T1D in biobreeding diabetes-prone (BB-DP) rats [124, 125]. Hyperpolarization of the mitochondrial inner membrane (MHP) is another major mitochondrial defect observed in CD4⁺ T cells in T1D [126]. MHP contributes to the pathogenesis of this disease through three mechanisms [126]. First, MHP reduces mitochondrial function, as evidenced by a reduction in the SRC, which impairs the ability of T cells to meet energy demands under stressful conditions. As a result, CD4⁺ T cells exhibit lower levels of intracellular ATP, which negatively affects their function. This lack of energy contributes to immune dysregulation, worsening the progression of T1D [124, 126, 127]. Second, MHP is associated with the differentiation of CD4⁺ T cells into the Th1 lineage, which is characterized by high production of IFN-γ. IFN-γ production sustains inflammatory responses and drives autoimmune destruction of pancreatic β-cells, a hallmark of T1D [124, 126–128]. Third, and most importantly, CD4⁺ T cells with MHP produce increased and unregulated amounts of mitochondrial ROS [124, 126, 128]. This increased ROS production is crucial for initiating and maintaining the metabolic reprogramming associated with T cell activation. ROS act as signaling molecules that promote mTOR signaling, subsequently increasing the expression of c-Myc and shifting cellular metabolism toward aerobic glycolysis [129]. This metabolic rearrangement in CD4⁺ T cells leads to enhanced activation of autoreactive T cells, which can directly damage β-cells. β-Cells are particularly vulnerable to damage because of their limited antioxidative capabilities, making them highly sensitive to oxidative stress. Therefore, the production of ROS and the subsequent metabolic changes in autoreactive T cells contribute to the autoimmune destruction of pancreatic β-cells in T1D [126, 130].

Thus, targeting ROS appears to be a promising therapeutic approach for T1D, as it could modulate CD4⁺ T cell metabolism and ultimately regulate CD4⁺ T cell activation. Manganese metalloporphyrin (MnP) is a powerful antioxidant that scavenges ROS and has the potential to modulate the immune and metabolic pathways involved in T1D [131]. MnP regulates ROS and influences redox-mediated regulation during T cell activation. This redox modulation results in decreased mTOR signaling, reduced glucose uptake, and decreased lactate production, ultimately shifting metabolism from OXPHOS to aerobic glycolysis in these cells. These metabolic changes limit CD4⁺ T cell growth and autoreactive activation, which are instrumental in mediating β-cell destruction in T1D. Furthermore, in adoptive transfer models, MnP treatment has been shown to delay the progression of diabetes, primarily by stabilizing mitochondrial function and limiting inflammatory responses [131]. The use of SOD mimetics has also been shown to delay the adoptive transfer of T1D cells with a diabetogenic CD4⁺ T cell clone, partly due to a decrease in T cell proliferation and IFN-γ production [132].

Together, these results demonstrate that mitochondrial dysfunction in CD4⁺ T cells plays a pivotal role in the pathogenesis of T1D. Targeting these defects leads to the regulation of CD4⁺ T cell metabolism and function, thereby mitigating the progression of T1D.

#### Dysregulated amino acid metabolism pathway in CD4^+^ T cells in T1D

The metabolism of amino acids, particularly glutamine, is dysregulated in T cells during T1D. Glutamine is decarboxylated to γ-aminobutyric acid (GABA) in T cells by the enzyme glutamic acid decarboxylase. GABA serves as an immunosuppressor because of its ability to block activation-induced calcium signaling and inhibit NF-κB activation. Together, these actions inhibit T cell activation and promote the induction of Tregs [133]. However, this process is dysfunctional or impaired in the context of T1D. Evidence has shown that low-dose GABA treatment in diabetes-prone NOD mice protects against insulitis by activating Tregs [134]. Furthermore, therapeutic approaches utilizing GABA or GABA-agonizing agents, such as benzodiazepines, have been shown to protect against T1D [133].

#### Dysregulated lipid metabolism in CD4^+^ T cells in T1D

Few studies have investigated changes in lipid metabolism in CD4⁺ T cells in patients with T1D. PBMCs isolated from patients with T1D exhibit increased lipid accumulation and elevated levels of lipid peroxidation upon exposure to fatty acids, indicating dysregulated lipid metabolism and oxidative stress. These alterations can impair mitochondrial function and induce apoptosis, potentially disrupting immune regulation and contributing to the development of autoimmunity [135].

In summary, disruption of CD4⁺ T cell metabolism has emerged as a crucial event driving the autoimmune destruction of pancreatic β-cells in T1D. Autoreactive CD4⁺ T cells preferentially rely on the pathological dependence on aerobic glycolysis rather than OXPHOS, a process further exacerbated by MHP and overproduction of ROS. This metabolic state promotes Th1 and Th17 proinflammatory responses while inhibiting Treg cell function. Additionally, dysregulated lipid metabolism leads to increased levels of lipid peroxidation and lipid accumulation in PBMCs from T1D patients, further contributing to mitochondrial dysfunction and apoptosis. Therapeutic strategies targeting dysregulated metabolic pathways have shown efficacy in restoring metabolic homeostasis in T cells, potentially interrupting the autoimmune cascade that drives β-cell destruction and thereby attenuating the progression of T1D (Table 3).

**Table 3:** Targeted metabolic pathways in T1D

MOLECULES AND DRUGS	Target Metabolic Pathway	Effects	References
RAPAMYCIN	mTROC1 Glycolysis (↓).	Tregs expansion in T1D mouse model (↑). Autoreactive T cells in T1D mouse model (↓).	[120, 121]
METFORMIN	HIF-1a and c-myc Glycolysis (↓).	Th1 and Th17 differentiation invitro (↓). Treg development in vitro (↑). Amounts of Th1 and Th17 cells in the spleens of NOD mice (↓). Insulitis in NOD mice (↓).	[122, 123]
PFK15	PFKFB3 Glycolysis (↓).	PD-1/LAG-3 expression on CD4^+^ T cells from T1D patients in vitro (↑). Exhaustion of Diabetogenic CD4^+^ T cell Clones in NOD mice. Delays Onset of T1D (↑).	[119]
MANGANESE METALLOPORPHYRIN	ROS Oxidative stress (↓). Glycolysis (↓).	CD4^+^ T cell growth and autoreactive activation (↓). Progression of diabetes in mice (↓).	[131]
SOD MIMETICS	ROS Glycolysis (↓).	T cell proliferation (↓). IFN-ᵧ production (↓). Cytotoxic CD8^+^ T cell effector responses (↓).	[132, 139]
GABA AGONISTS	Normalized aa metabolism and GABA level	Treg activation (↑). Protects against insulitis.	[193]
2DG	HK Glycolysis (↓).	Diabetogenic CD8^+^ T cells in the islets of T1D mouse model. Delayed T1D onset (↓). Protected ß-cell mass.	[138]

*mTORC1* Mechanistic target of rapamycin complex 1, *HIF-1a* Hypoxia-inducible factor 1-alpha, *PFKFB3* 6-Phosphofructo-2-kinase/fructose-2,6-bisphosphatase 3, *ROS* Reactive oxygen species, *SOD* Superoxide dismutase, *IFN-γ* Interferon gamma, *GABA* Gamma-aminobutyric acid, *aa* Amino acid, *2DG* 2-deoxy-D-glucose, *HK* Hexokinase

### Dysregulation of the glycolytic pathway and mitochondrial dysfunction in CD8^+^ T cells in T1D

In T1D, CD8^+^ T cells, along with CD4^+^ T cells, contribute to the destruction of pancreatic β-cells. The release of IFN-γ by Th1 cells activates autoreactive, cytotoxic CD8^+^ T cells, enhancing their cytolytic activity through granzyme/perforin-mediated mechanisms and Fas/FasL interactions, which induce apoptosis in β-cells. This creates a destructive feedback loop, wherein activated CD8^+^ T cells recruit innate immune cells and amplify the inflammatory cascade by producing cytokines that contribute to insulitis [3, 136]. Although most metabolic research has focused on CD4^+^ T cells, emerging evidence suggests that CD8^+^ T cells also undergo pathogenic metabolic reprogramming in T1D (Fig. 6B).

In T1D, autoreactive CD8⁺ T cells play a central role in destroying pancreatic β cells, and recent work has revealed that their metabolism is a key factor in this pathogenic process. In a NOD mouse model, researchers identified a population of islet antigen-specific CD8⁺ T cells that recognize a specific epitope [137]. These diabetogenic T cells accumulate with age and display an antigen-experienced phenotype characterized by high CD44 expression, low CD62L expression, and high PD-1 and CXCR3 expression, which is consistent with a memory precursor-like state rather than a fully differentiated effector state. Despite this semidormant phenotype, they retain strong pathogenic potential. Transcriptomic and functional analyses revealed that these autoreactive CD8⁺ T cells exhibit altered metabolic programming compared with that of conventional effector T cells [138]. Specifically, they display a reduced dependence on mitochondrial OXPHOS and an increased reliance on glycolytic metabolism for survival and function. In vivo inhibition of glycolysis with 2DG selectively decreased the frequency of these diabetogenic T cells, decreased islet infiltration, and preserved β-cell granularity, indicating that their pathogenic activity depends on sustained glucose metabolism [119, 138]. This metabolic pattern is OXPHOS^low^ and glycolytic^high^ dependent and differs from that of typical long-lived memory T cells, which usually rely on FAO, suggesting that diabetogenic CD8⁺ T cells occupy a unique hybrid metabolic state. Such a profile may allow them to persist chronically while remaining poised for cytotoxic activity in the nutrient-fluctuating environment of the islet. Importantly, these findings link cellular metabolism directly to autoimmunity and suggest that targeting glycolysis or other metabolic pathways could selectively suppress autoreactive T cells without broadly compromising immune defense. Thus, the altered metabolic programming of diabetogenic CD8⁺ T cells not only contributes to the persistence and pathogenicity of autoimmunity in T1D but also offers a promising avenue for therapeutic intervention through metabolic modulation [138].

Human CD8^+^ T cells from individuals with T1D also display MHP, which increases their cytotoxicity against human β-cells [124]. Moreover, elevated ROS levels have been observed in CD8⁺ T cells, and reducing ROS levels or glycolytic activity through the upregulation of SOD expression has been shown to suppress cytotoxic CD8⁺ T cell effector functions, potentially delaying the onset of T1D [139].

In summary, although CD8⁺ T cells play a central role in the pathogenesis of T1D and exhibit distinct immunometabolic alterations, their metabolic pathways remain incompletely understood and are only beginning to be elucidated.

### Dysregulated glycolytic pathway activity and lipid metabolism in B cells in T1D

While destruction of pancreatic β-cells by autoreactive T cells is considered the major cause of immunopathogenesis in T1D, recent findings have increasingly highlighted the critical role of B lymphocytes in the progression of this disease, but few studies have investigated B cell metabolism in T1D (Fig. 6C). B cells, in particular, contribute through their ability to express costimulatory molecules and present antigens, which are essential for the activation of autoreactive T lymphocytes. There is growing evidence that the processes of B cell development, differentiation, function, and metabolism significantly influence the onset and progression of T1D. Research has indicated that NOD mice with B cell deficiencies are notably resistant to the development of T1D [140]. Furthermore, B cell depletion has the potential to restore the functionality of pancreatic β-cells, underscoring their role in the pathogenesis of T1D [141].

A recent study revealed that high-glucose-avidity B cells with inflammatory functions are increased in both T1D patients and NOD mice. These high-glucose-avidity B cells exhibited increased Myc expression, which in turn upregulated the expression of glycolysis-related genes such as GLUT1, HK2, and LDHA and increased the production of inflammatory cytokines. These metabolic shifts through increasing the activity of the glycolytic pathway are associated with B cell activation, autoantibody production, and broader immune modulation. Compared with those from healthy controls, peripheral B cells isolated from patients with T1D consistently exhibit increased expression of glycolytic genes [142, 143]. These findings indicate that c-Myc serves as a crucial transcription factor in modulating the glycolysis-related inflammatory characteristics of B cells in both T1D patients and NOD mice.

Targeting glucose metabolism through the use of metformin and 2DG in a diabetic mouse model reduced the pathogenic phenotype of B cells and provided protection against the onset of T1D [142].

Another study also revealed that B cells from NOD mice exhibit higher glucose uptake than those from nondiabetic mice do. Additionally, HIF-1α-driven glycolytic flux is insufficient in B cells from NOD mice. With respect to the crucial role of HIF-1α in regulatory B cell development, its dysfunction in NOD mice results in impaired regulatory B cell formation, potentially driving disease progression [144].

In addition to metabolic dysregulation in glucose metabolism, alterations in lipid metabolism have been observed in B cells from NOD mice. These B cells exhibit increased expression of CD36, a fatty acid transporter, which leads to increased lipid uptake in autoreactive B cells as an energy source [145]. The increased affinity for lipid metabolites, attributed to the upregulation of CD36, may influence B cell functionality. This elevated lipid uptake enhances B cell metabolism, fueling proliferation and plasma cell differentiation, thereby exacerbating autoreactive responses in T1D pathogenesis [140].

In conclusion, B cell metabolism plays a substantial role in the immunopathogenesis of T1D, contributing to both the initiation and progression of the disease. Dysregulated glucose and lipid metabolism in B cells, characterized by increased glycolytic activity and enhanced lipid uptake via CD36, contributes to their pathogenic function in T1D. Targeting metabolic pathways, such as Myc-driven glycolysis and lipid metabolism, offers a potential therapeutic approach to modulating B cell function and mitigating T1D progression (Table 3).

### Integrated metabolic signature of lymphocytes in T1D

Metabolic reprogramming plays a central role in the pathogenesis of T1D by providing the energy needed for autoimmune-mediated destruction of insulin-secreting β cells [140]. One characteristic feature of pathogenic CD4⁺ and CD8⁺ T cells is increased utilization of glycolysis, which enables rapid proliferation, inflammatory cytokine production, and long-term effector function [119, 138]. This process is regulated at the upstream level by factors such as mTOR, HIF-1α, and c-Myc, as well as by increased mitochondrial ROS levels, which act as signals that potentiate inflammatory responses. Notably, these metabolic alterations both increase the efficacy of pathogenic T cells and disrupt the formation of tolerogenic Tregs [146].

Mitochondrial dysfunction is yet another key element of metabolic dysfunction in T1D [126, 147]. Hyperpolarization of the mitochondrial membrane, reduced OXPHOS efficiency, and ROS generation affect T cell activity and viability [124, 126]. These changes in mitochondria help increase inflammation and make autoreactive cells more efficient at killing pancreatic β cells [148]. Moreover, problems in amino acid metabolism and specifically in the metabolism of glutamine, which regulates immunity, decrease anti-inflammatory signaling, such as that involving GABA, making autoimmunity even more likely [149].

Metabolic rewiring has also been noted in B cells from T1D patients, as evidenced by increases in glucose intake, glycolytic gene expression, and lipid intake [142]. All these factors aid in the stimulation of B cells, the generation of autoantibodies, and the presentation of antigens to induce stronger responses from autoreactive T cells. Together, these findings help to elucidate how disruption of glycolysis, mitochondria, and nutrient metabolism can facilitate constant immune system activation and β-cell death. Notably, metabolic changes are similar in many other autoimmune diseases.

## Systemic lupus erythematosus

SLE is an example of a systemic autoimmune disorder involving the disruption of immune tolerance, the presence of autoantibodies, and inflammation in many tissues [150]. Despite being generally categorized as a B cell-dependent disorder, T cells are vital for the induction of autoimmunity through helper functions for autoreactive B cells and the generation of proinflammatory cytokines [151]. Mounting evidence has shown that metabolic regulation in T and B cells may influence SLE development [152].

### Dysregulated T cell metabolism in SLE

Autoreactive CD4⁺ T cells in SLE exhibit profound metabolic alterations characterized by increased glycolysis and mitochondrial dysfunction [153]. Elevated glycolytic activity supports T cell activation, proliferation, and the production of inflammatory cytokines such as IFN-γ and IL-17. This glycolytic shift is driven by the activation of key metabolic regulators, including mTOR and HIF-1α [154]. Increased mTOR signaling has been strongly implicated in promoting inflammatory T cell differentiation while impairing Treg function, thereby contributing to the loss of immune tolerance [155].

Mitochondrial alterations also constitute another characteristic feature of SLE T cells. SLE T cells are characterized by hyperpolarized mitochondria, mitochondrial hypertrophy, and mitochondrial ROS overproduction [156]. ROS play a role as signaling molecules for further inflammation-induced upregulation of T cell activation [157, 158]. On the other hand, mitochondrial dysregulation causes decreased energy production and disruption of metabolic balance [159]. Of great interest is the evidence provided that demonstrates the effectiveness of rapamycin treatment in the normalization of T cell metabolism and SLE disease activity, highlighting the vital importance of metabolic pathways for the development of SLE [160].

**Fig. 7:**
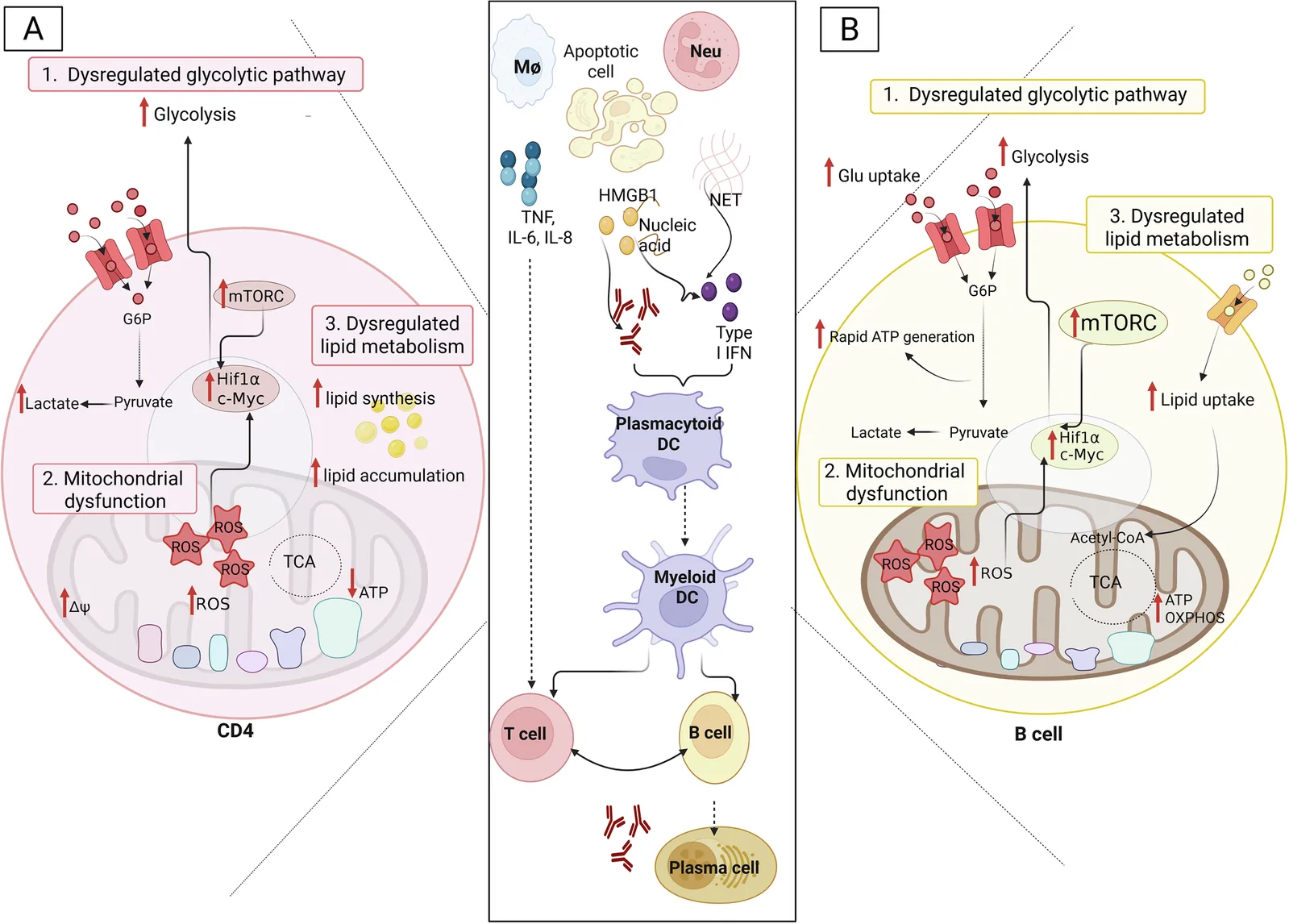
Metabolic alterations in T cells and B cells in SLE. **A** In SLE, CD4⁺ T cells display enhanced glycolytic activity, driving a metabolic shift toward glycolysis that is essential for their activation, proliferation, and pro-inflammatory cytokine production while impairing Treg function. These cells exhibit mitochondrial alterations, including MHP, elevated mitochondrial mass, and heightened ROS generation, which collectively promote HIF-1α and c-Myc induction and further reinforce glycolytic reprogramming. Upregulated lipogenesis contributes to membrane remodeling and supports sustained activation. **B** B cells in SLE similarly depend on glycolysis to sustain their increased proliferative capacity and autoantibody production. Mitochondrial dysfunction, accompanied by elevated ROS levels and aberrant OXPHOS activity, facilitates plasma cell differentiation and augments inflammatory responses. Dysregulated lipid metabolism, particularly increased FAO, is associated with enhanced B cell survival. The figure was created with BioRender software https://www.biorender.com

Alterations in lipid metabolism also contribute to T cell dysfunction in patients with SLE. Increased lipid synthesis and accumulation promote membrane remodeling and support inflammatory signaling pathways (Fig. 7A). In addition, lipid metabolites can directly influence T cell differentiation and cytokine production, further amplifying immune dysregulation [161, 162].

### Dysregulated B cell metabolism in SLE

SLE involves marked metabolic changes in B cells, which drive their disease-causing activities. Specifically, autoreactive B cells are characterized by high glucose uptake and increased glycolysis to provide the necessary substrates for their survival, proliferation, cytokine synthesis, and secretion of autoantibodies [163, 164]. The switch to glycolysis allows the rapid production of ATP to increase metabolic activity; however, the accumulation of glycolytic intermediates may result in the formation of ROS that induce inflammation and damage tissues. In addition to glycolytic stimulation, activated B lymphocytes in SLE show increased mitochondrial respiration and OXPHOS, which aid in the differentiation of B cells into antibody-producing PCs [165]. Metabolic rewiring in B lymphocytes could also favor the survival of autoreactive B cells that continue to produce antibodies. In addition, B cells in patients with SLE exhibit changes in fatty acid metabolism in which the utilization of fatty acids is upregulated [166]. This may be useful for B cell survival during states of cellular stress, allowing them to survive and remain functional in the production of antibodies during the autoimmune response. Importantly, metabolic signaling cascades such as mTOR and c-Myc play important roles in the regulation of B cell activation and differentiation in patients with SLE [167]. Metabolically active B cells can maintain their ability to produce autoantibodies and induce chronic inflammation (Fig. 7B).

### Integrated metabolic signature of lymphocytes in SLE

Overall, there is sufficient evidence to support that metabolic rewiring is a key characteristic of abnormal lymphocyte functions in patients with SLE. For example, autoreactive T cells exhibit high levels of glycolysis, mitochondrial malfunction, and excessive production of ROS [156, 168]. These metabolic changes enhance inflammation while inhibiting immune tolerance [155, 157]. On the other hand, metabolically active B cells depend on efficient glucose metabolism and mitochondria to generate autoantibodies and autoimmune reactions [164].

The important aspect of immunometabolic regulation in the pathology of SLE is the upregulation of metabolic regulators such as mTOR signaling, which is used by the cell to coordinate environmental and cellular cues to determine the fate of the cell [169]. The continuous activation of the pathway results in inflammation and differentiation of lymphocytes while inhibiting regulatory processes. Treatment modalities directed at regulating metabolic pathways have been proven to be beneficial in clinical settings [160, 170]. These findings support the involvement of immunometabolic dysregulation as a central regulator of this disease.

## Immunoregulatory role of metabolites in autoimmune diseases

Metabolites serve a dual purpose as both energy suppliers and crucial regulators of immune cell function and differentiation [171]. A growing body of evidence confirms that the formation of defective metabolites may directly influence immune responses and autoimmune disorders [172]. In this context, SCFA such as butyric acid have become important immunomodulators. Butyric acid, which is derived mainly from the gut microflora, plays an immunosuppressive role by promoting Treg differentiation via epigenetic regulation through the inhibition of histone deacetylase activity and gene expression [173]. Moreover, butyrate inhibits the generation of proinflammatory cytokines and Th17 cell development, which helps reduce autoimmune-induced inflammation [174]. Low levels of butyrate have been reported in some autoimmune disorders, such as MS and T1D, suggesting that reduced butyrate formation plays a role in disease pathogenesis [57, 175].

Other metabolites, such as gluconolactone, have critical regulatory functions because of their ability to regulate the redox state of cells and control metabolic flow through the PPP [176]. Gluconolactone controls the synthesis of NADPH, an important metabolic intermediate that is needed to maintain redox balance and reduce reactive oxygen species. An imbalance in this process leads to the development of oxidative stress and proinflammatory signaling in immune cells [177]. Additionally, metabolites such as lactate, succinate, and fatty acids act as signaling molecules [178–180].

Most importantly, the results demonstrate that metabolites play important roles in the regulation of the immune system rather than being mere products of metabolism. Metabolite imbalance, which might be caused by abnormal cell metabolism or environmental conditions, such as changes in the microflora, affects the maintenance of immune homeostasis and leads to the development of autoimmune diseases [172].

## Regulation of metabolic enzymes by kinases and transcription factors in autoimmune lymphocytes

Metabolic reprogramming in autoimmune lymphocytes is not solely driven by substrate availability but is tightly regulated by signaling pathways that control the activity and expression of metabolic enzymes [181]. Central to this regulation are protein kinases that modify metabolic enzymes through phosphorylation, thereby rapidly altering their activity [182]. mTOR serves as the master regulator of metabolism and coordinates the availability of nutrients, growth factors, and inflammatory signals [183]. Activation of the mTORC1 complex facilitates increased glucose metabolism by inducing the translation of important metabolic proteins while simultaneously increasing glucose entry into cells. On the other hand, AMPK is an energy stress sensor that inhibits anabolic processes and induces oxidative metabolism [184]. Dysregulated mTOR‒AMPK signaling has been consistently observed in autoimmune diseases and contributes to sustained effector T cell responses and impaired Treg function [185].

Kinase signaling is not the only means by which transcription factors can regulate metabolism. HIF-1α induces the expression of glycolytic enzymes such as hexokinase and lactate dehydrogenase, promotes Th17 cell development, and suppresses Tregs [186]. The transcription factor c-Myc is responsible for the regulation of genes associated with glycolysis, glutaminolysis, and nucleotide synthesis to ensure rapid proliferation of activated lymphocytes. The transcription factors STAT3 and NF-κB are additional mediators that link inflammation with metabolism [187].

Importantly, kinase-driven phosphorylation events and transcription factor-mediated gene regulation act in concert to shape metabolic enzyme activity and cellular fate decisions. Persistent activation of these regulatory networks in autoimmune diseases promotes anabolic metabolism, inflammatory cytokine production, and the survival of autoreactive lymphocytes. Thus, the aberrant control of metabolic enzymes by kinases and transcription factors represents a central mechanistic link between immune signaling and metabolic dysfunction in autoimmunity.

## Shared immunometabolic mechanisms across autoimmune diseases

Although MS, RA, T1D and SLE affect different target tissues, accumulating evidence indicates that these diseases share fundamental immunometabolic abnormalities that drive lymphocyte pathogenicity. A central and recurring feature is the reprogramming of glucose metabolism in autoreactive lymphocytes. Increased glycolysis supports rapid proliferation, cytokine production, and differentiation of inflammatory T cell subsets, particularly Th1 and Th17 cells, whereas Tregs exhibit impaired metabolic fitness [188]. However, in other instances, for example, during RA, glucose metabolism is channeled into the PPP, which favors anabolic activity and inflammation [189]. These results demonstrate the critical role of impaired glucose metabolism in immune homeostasis.

Impairment in mitochondrial activity is yet another important metabolic characteristic seen in various autoimmune conditions. Impaired OXPHOS, mitochondrial hyperpolarization, and excessive generation of ROS lead to prolonged immune stimulation and autoreactive lymphocyte survival [190]. Additionally, mitochondria-derived signals play a role in controlling inflammatory gene expression and lymphocyte differentiation. In tandem, alterations in lipid metabolism and the availability of immunomodulatory metabolites modify the immune response through inflammation induction and regulatory dysfunction [191]. These metabolic alterations affect not only T cells but also B cells, enhancing their activation, antigen presentation, and autoantibody production (Table 4).

**Table 4:** Shared and disease-specific immunometabolic alterations in autoimmune diseases

METABOLIC PATHWAY / FEATURE	Multiple Sclerosis (MS)	Rheumatoid Arthritis (RA)	Type 1 Diabetes (T1D)	Systemic Lupus Erythematosus (SLE)	Functional Consequence
GLYCOLYSIS	Increased glycolysis in CD4^+^ T cells; promotes Th17 differentiation	Reduced glycolysis, glucose diverted to PPP in CD4^+^ T cells	Increased glycolysis in autoreactive CD4^+^ and CD8^+^ T cells	Increased glycolysis in CD4^+^ T cells and autoreactive B cells	Supports proliferation, inflammatory cytokine production, and effector differentiation
PENTOSE PHOSPHATE PATHWAY (PPP)	Moderately increased	Strongly increased; excess NADPH production	Increased	Increased	Promotes anabolic metabolism, redox imbalance, and cell survival
MITOCHONDRIAL FUNCTION	Mitochondrial dysfunction, increased ROS	Impaired oxidative phosphorylation, mitochondrial abnormalities	Mitochondrial hyperpolarization, increased ROS	Mitochondrial hyperpolarization, increased ROS	Enhances inflammatory signaling and prolongs survival of autoreactive lymphocytes
REACTIVE OXYGEN SPECIES (ROS)	Increased mitochondrial ROS promotes Th17 responses	Increased oxidative stress	Increased mitochondrial ROS	Excessive ROS production	Amplifies inflammatory pathways and immune activation
LIPID METABOLISM	Increased fatty acid synthesis; altered fatty acid oxidation	Increased lipid synthesis and accumulation	Increased lipid uptake in B cells	Increased lipid synthesis	Supports membrane formation, proliferation, and inflammatory signaling
B-CELL METABOLISM	Increased oxidative phosphorylation and activation	Increased glycolysis and metabolic activation	Increased glucose uptake and lipid metabolism	Increased glycolysis and mitochondrial metabolism	Promotes autoantibody production and antigen presentation
KEY METABOLIC REGULATORS	mTOR, HIF-1α, c-Myc, AMPK	mTOR, AMPK, ATM	mTOR, HIF-1α, c-Myc	mTOR, HIF-1α	Control metabolic programming and immune cell differentiation
KEY IMMUNOMODULATORY METABOLITES	Lactate, fatty acids, propionate	Lactate	ROS, glutamine metabolites	ROS, lipids	Regulate immune cell activation and inflammation
THERAPEUTIC METABOLIC TARGETING	Dimethyl fumarate, metabolic inhibitors	mTOR inhibitors (experimental)	Metabolic modulation (experimental)	Rapamycin (mTOR inhibitor) effective	Restores immune balance and reduces inflammation

These similar metabolic functions are strictly controlled through a signaling pathway composed of mTOR, AMPK, HIF-1α, and c-Myc, which respond to environmental signals and integrate metabolic programming within immune cells to dictate cell fate. The prolonged activation of metabolic pathways results in inflammation, whereas abnormal metabolism leads to immune dysregulation. Taken together, this evidence suggests a model whereby coordinated metabolic repurposing can lead to dysregulated immunity in autoimmune disorders. This knowledge is valuable for understanding the underlying mechanism involved in the development of these diseases, with immunometabolism as a potential treatment strategy.

## Future directions

Despite great progress, there are a number of challenges to overcome to turn these findings into effective therapeutic strategies against autoimmunity. One such challenge is represented by the new opportunities for studying the metabolism of individual cells provided by the recent introduction of single-cell metabolomics. The metabolic state of autoreactive and regulatory lymphocytes is largely unknown because of bulk-based analysis, which obscures critical differences between cell populations and their dynamics during disease progression and treatment. The combination of single-cell transcriptomic, epigenetic, and metabolic profiling should allow the identification of disease-causing clones and the dynamic study of their metabolic changes. Another challenge that needs to be addressed is metabolic heterogeneity in lymphocyte populations. Although apparently similar, different subsets have distinct metabolic signatures depending on tissue localization, exposure to inflammatory signals, and available nutrients. Identifying how microenvironmental cues contribute to metabolic diversity may facilitate discrimination between protective and pathogenic immune responses. Target specificity is another crucial aspect, as targeting ubiquitous metabolic processes leads to generalized immunosuppression and toxicity. Research on the metabolic vulnerabilities of autoreactive lymphocytes, which could be used for designing novel, highly specific drugs and delivering them to specific tissues, should therefore receive priority. Furthermore, clinical translation requires establishing the role of metabolic markers in human disease and validating potential metabolic biomarkers in autoimmunity. Metabolic biomarkers could be used to stratify patients, assess therapeutic efficacy, and develop more personalized immunometabolic treatments. However, some questions have not yet been answered. Specifically, whether metabolism drives the initiation or sustenance of autoimmunity, how stable and reversible metabolic changes are, and whether it would be possible to reprogram them as a treatment strategy are unknown.

## Conclusions

Understanding the metabolic alterations in lymphocytes—particularly the shift from OXPHOS to aerobic glycolysis, fatty acid synthesis, and glutaminolysis—during T cell-mediated autoimmunity provides critical insights into disease pathogenesis and reveals new opportunities for therapeutic intervention. In T1D, autoreactive CD4⁺ T cells depend on glycolysis to sustain insulitis, whereas in MS, dysregulated Th17 and B cells leverage metabolic reprogramming to drive CNS inflammation. Similarly, in RA, synovial T and B cells adopt hypermetabolic states that amplify joint destruction. These shared mechanisms highlight immune metabolism as a central driver of autoimmunity, transcending disease-specific contexts.

Targeting these pathways with glycolysis inhibitors (e.g., 2DG), mTOR inhibitors (e.g., rapamycin), or other metabolic reprogramming strategies could disrupt pathogenic immune responses and restore homeostasis. However, challenges remain in achieving cell-specific targeting and avoiding systemic toxicity.

## References

[1] Yasmeen F, Pirzada RH, Ahmad B, Choi B, Choi S. Understanding Autoimmunity: Mechanisms, Predisposing Factors, and Cytokine Therapies. Int J Mol Sci. 2024;25:7666. 10.3390/ijms25147666 PMC11277571 39062908

[2] Weiner HL. Multiple sclerosis is an inflammatory T-cell-mediated autoimmune disease. Arch Neurol. 2004;61:1613–5. 10.1001/archneur.61.10.1613 15477521

[3] Burrack AL, Martinov T, Fife BTT. Cell-Mediated Beta Cell Destruction: Autoimmunity and Alloimmunity in the Context of Type 1 Diabetes. Front Endocrinol (Lausanne). 2017;8:343. 10.3389/fendo.2017.00343 PMC5723426 29259578

[4] Carlé C, Degboe Y, Ruyssen-Witrand A, Arleevskaya MI, Clavel C, Renaudineau Y. Characteristics of the (Auto)Reactive T Cells in Rheumatoid Arthritis According to the Immune Epitope Database. Int J Mol Sci. 2023;24:4296. 10.3390/ijms24054296 PMC10001542 36901730

[5] Galgani M, Bruzzaniti S, Matarese G. Immunometabolism and autoimmunity. Curr Opin Immunol. 2020;67:10–7. 10.1016/j.coi.2020.07.002 32784085

[6] Al-Khami AA, Rodriguez PC, Ochoa AC. Energy metabolic pathways control the fate and function of myeloid immune cells. J Leukoc Biol. 2017;102:369–80. 10.1189/jlb.1VMR1216-535R PMC5505747 28515225

[7] Pavlova NN, Thompson CB. The Emerging Hallmarks of Cancer Metabolism. Cell Metab. 2016;23:27–47. 10.1016/j.cmet.2015.12.006 PMC4715268 26771115

[8] Martínez-Reyes I, Chandel NS, Mitochondrial TCA. Mitochondrial TCA cycle metabolites control physiology and disease. Nat Commun. 2020;11:102. 10.1038/s41467-019-13668-3 PMC6941980 31900386

[9] Vercellino I, Sazanov LA. The assembly, regulation and function of the mitochondrial respiratory chain. Nat Rev Mol Cell Biol. 2022;23:141–61. 10.1038/s41580-021-00415-0 34621061

[10] Wilson DF. Oxidative phosphorylation: regulation and role in cellular and tissue metabolism. J Physiol. 2017;595:7023–38. 10.1113/JP273839 PMC5709332 29023737

[11] Almeida L, Lochner M, Berod L, Sparwasser T. Metabolic pathways in T cell activation and lineage differentiation. Semin Immunol. 2016;28:514–24. 10.1016/j.smim.2016.10.009 27825556

[12] Wofford JA, Wieman HL, Jacobs SR, Zhao Y, Rathmell JC. IL-7 promotes Glut1 trafficking and glucose uptake via STAT5-mediated activation of Akt to support T-cell survival. Blood. 2008;111:2101–11. 10.1182/blood-2007-06-096297 PMC2234050 18042802

[13] Yang Z, Matteson EL, Goronzy JJ, Weyand CM. T-cell metabolism in autoimmune disease. Arthritis Res Ther. 2015;17:29. 10.1186/s13075-015-0542-4 PMC4324046 25890351

[14] Pearce EL. Metabolism in T cell activation and differentiation. Curr Opin Immunol. 2010;22:314–20. 10.1016/j.coi.2010.01.018 PMC4486663 20189791

[15] Wang R, Dillon CP, Shi LZ, Milasta S, Carter R, Finkelstein D, et al. The transcription factor Myc controls metabolic reprogramming upon T lymphocyte activation. Immunity. 2011;35:871–82. 10.1016/j.immuni.2011.09.021 PMC3248798 22195744

[16] Macintyre AN, Gerriets VA, Nichols AG, Michalek RD, Rudolph MC, Deoliveira D, et al. The glucose transporter Glut1 is selectively essential for CD4 T cell activation and effector function. Cell Metab. 2014;20:61–72. 10.1016/j.cmet.2014.05.004 PMC4079750 24930970

[17] Buck MD, O’Sullivan D, Pearce EL. T cell metabolism drives immunity. J Exp Med. 2015;212:1345–60. 10.1084/jem.20151159 PMC4548052 26261266

[18] Berod L, Friedrich C, Nandan A, Freitag J, Hagemann S, Harmrolfs K, et al. De novo fatty acid synthesis controls the fate between regulatory T and T helper 17 cells. Nat Med. 2014;20:1327–33. 10.1038/nm.3704 25282359

[19] Ren W, Liu G, Yin J, Tan B, Wu G, Bazer FW, et al. Amino-acid transporters in T-cell activation and differentiation. Cell Death Dis. 2017;8:e2655. 10.1038/cddis.2016.222 PMC5386510 28252650

[20] Johnson MO, Wolf MM, Madden MZ, Andrejeva G, Sugiura A, Contreras DC, et al. Distinct Regulation of Th17 and Th1 Cell Differentiation by Glutaminase-Dependent Metabolism. Cell. 2018;175:1780–95.e19. 10.1016/j.cell.2018.10.001 PMC6361668 30392958

[21] Tomaszewicz M, Ronowska A, Zieliński M, Jankowska-Kulawy A, Trzonkowski P. T regulatory cells metabolism: The influence on functional properties and treatment potential. Front Immunol. 2023;14:1122063. 10.3389/fimmu.2023.1122063 PMC10081158 37033990

[22] Rieusset J. The role of endoplasmic reticulum-mitochondria contact sites in the control of glucose homeostasis: an update. Cell Death Dis. 2018;9:388. 10.1038/s41419-018-0416-1 PMC5844895 29523782

[23] Rangel Rivera GO, Knochelmann HM, Dwyer CJ, Smith AS, Wyatt MM, Rivera-Reyes AM, et al. Fundamentals of T Cell Metabolism and Strategies to Enhance Cancer Immunotherapy. Front Immunol. 2021;12:645242. 10.3389/fimmu.2021.645242 PMC8014042 33815400

[24] Victora GD, Nussenzweig MC. Germinal Centers. Annu Rev Immunol. 2022;40:413–42. 10.1146/annurev-immunol-120419-022408 35113731

[25] Weisel FJ, Mullett SJ, Elsner RA, Menk AV, Trivedi N, Luo W, et al. Germinal center B cells selectively oxidize fatty acids for energy while conducting minimal glycolysis. Nat Immunol. 2020;21:331–42. 10.1038/s41590-020-0598-4 PMC7112716 32066950

[26] Ripperger TJ, Bhattacharya D. Transcriptional and Metabolic Control of Memory B Cells and Plasma Cells. Annu Rev Immunol. 2021;39:345–68. 10.1146/annurev-immunol-093019-125603 33556247

[27] Chen Y, Gaber T. Hypoxia/HIF Modulates Immune Responses. Biomedicines. 2021;9:260. 10.3390/biomedicines9030260 PMC8000289 33808042

[28] Dominguez-Sola D, Victora GD, Ying CY, Phan RT, Saito M, Nussenzweig MC, et al. The proto-oncogene MYC is required for selection in the germinal center and cyclic reentry. Nat Immunol. 2012;13:1083–91. 10.1038/ni.2428 PMC3711534 23001145

[29] Calado DP, Sasaki Y, Godinho SA, Pellerin A, Köchert K, Sleckman BP, et al. The cell-cycle regulator c-Myc is essential for the formation and maintenance of germinal centers. Nat Immunol. 2012;13:1092–100. 10.1038/ni.2418 PMC4132664 23001146

[30] Shiraz AK, Panther EJ, Reilly CM. Altered Germinal-Center Metabolism in B Cells in Autoimmunity. Metabolites. 2022;12:40. 10.3390/metabo12010040 PMC8780703 35050162

[31] Ghasemi N, Razavi S, Nikzad E. Multiple Sclerosis: Pathogenesis, Symptoms, Diagnoses and Cell-Based Therapy. Cell J. 2017;19:1–10. 10.22074/cellj.2016.4867 PMC5241505 28367411

[32] Shi LZ, Wang R, Huang G, Vogel P, Neale G, Green DR, et al. HIF1alpha-dependent glycolytic pathway orchestrates a metabolic checkpoint for the differentiation of TH17 and Treg cells. J Exp Med. 2011;208:1367–76. 10.1084/jem.20110278 PMC3135370 21708926

[33] Seki SM, Stevenson M, Rosen AM, Arandjelovic S, Gemta L, Bullock TNJ, et al. Lineage-Specific Metabolic Properties and Vulnerabilities of T Cells in the Demyelinating Central Nervous System. J Immunol. 2017;198:4607–17. 10.4049/jimmunol.1600825 PMC5485845 28507026

[34] Mangan PR, Harrington LE, O’Quinn DB, Helms WS, Bullard DC, Elson CO, et al. Transforming growth factor-beta induces development of the T(H)17 lineage. Nature. 2006;441:231–4. 10.1038/nature04754 16648837

[35] Zhang D, Jin W, Wu R, Li J, Park SA, Tu E, et al. High Glucose Intake Exacerbates Autoimmunity through Reactive-Oxygen-Species-Mediated TGF-β Cytokine Activation. Immunity. 2019;51:671–81.e5. 10.1016/j.immuni.2019.08.001 PMC9811990 31451397

[36] Wu L, Hollinshead KER, Hao Y, Au C, Kroehling L, Ng C, et al. Niche-Selective Inhibition of Pathogenic Th17 Cells by Targeting Metabolic Redundancy. Cell. 2020;182:641–54.e20. 10.1016/j.cell.2020.06.014 PMC7556360 32615085

[37] Vakili ME, Shah Mohammadi FN, Ataollahi MR, Shams K, Eklund KK, Daryabor G, et al. Targeting T Cell Metabolism as a Novel Approach for Treatment of MS: With a Focus on PFKFB3 Inhibitors. Endocr Metab Immune Disord Drug Targets. 2023;23:417–22. 10.2174/1871530322666220921160930 36154602

[38] Sun Y, Tian T, Gao J, Liu X, Hou H, Cao R, et al. Metformin ameliorates the development of experimental autoimmune encephalomyelitis by regulating T helper 17 and regulatory T cells in mice. J Neuroimmunol. 2016;292:58–67. 10.1016/j.jneuroim.2016.01.014 26943960

[39] Liebmann M, Hucke S, Koch K, Eschborn M, Ghelman J, Chasan AI, et al. Nur77 serves as a molecular brake of the metabolic switch during T cell activation to restrict autoimmunity. Proc Natl Acad Sci USA. 2018;115:E8017–e26. 10.1073/pnas.1721049115 PMC6112725 30072431

[40] Yang G, Song W, Postoak JL, Chen J, Martinez J, Zhang J, et al. Autophagy-related protein PIK3C3/VPS34 controls T cell metabolism and function. Autophagy. 2021;17:1193–204. 10.1080/15548627.2020.1752979 PMC8143264 32268825

[41] De Riccardis L, Ferramosca A, Danieli A, Trianni G, Zara V, De Robertis F, et al. Metabolic response to glatiramer acetate therapy in multiple sclerosis patients. BBA Clin. 2016;6:131–7. 10.1016/j.bbacli.2016.10.004 PMC5079236 27785417

[42] Zahoor I, Suhail H, Datta I, Ahmed ME, Poisson LM, Waters J, et al. Blood-based untargeted metabolomics in relapsing-remitting multiple sclerosis revealed the testable therapeutic target. Proc Natl Acad Sci USA. 2022;119:e2123265119. 10.1073/pnas.2123265119 PMC9231486 35700359

[43] Greeck VB, Williams SK, Haas J, Wildemann B, Fairless R. Alterations in Lymphocytic Metabolism-An Emerging Hallmark of MS Pathophysiology? Int J Mol Sci. 2023;24:2094. 10.3390/ijms24032094 PMC9917089 36768415

[44] Gonzalo H, Nogueras L, Gil-Sánchez A, Hervás JV, Valcheva P, González-Mingot C, et al. Impairment of Mitochondrial Redox Status in Peripheral Lymphocytes of Multiple Sclerosis Patients. Front Neurosci. 2019;13:938. 10.3389/fnins.2019.00938 PMC6738270 31551694

[45] De Riccardis L, Rizzello A, Ferramosca A, Urso E, De Robertis F, Danieli A, et al. Bioenergetics profile of CD4(+) T cells in relapsing remitting multiple sclerosis subjects. J Biotechnol. 2015;202:31–9. 10.1016/j.jbiotec.2015.02.015 25701681

[46] La Rocca C, Carbone F, De Rosa V, Colamatteo A, Galgani M, Perna F, et al. Immunometabolic profiling of T cells from patients with relapsing-remitting multiple sclerosis reveals an impairment in glycolysis and mitochondrial respiration. Metabolism. 2017;77:39–46. 10.1016/j.metabol.2017.08.011 PMC5800394 29132538

[47] De Rosa V, Galgani M, Porcellini A, Colamatteo A, Santopaolo M, Zuchegna C, et al. Glycolysis controls the induction of human regulatory T cells by modulating the expression of FOXP3 exon 2 splicing variants. Nat Immunol. 2015;16:1174–84. 10.1038/ni.3269 PMC4868085 26414764

[48] Klotz L, Eschborn M, Lindner M, Liebmann M, Herold M, Janoschka C, et al. Teriflunomide treatment for multiple sclerosis modulates T cell mitochondrial respiration with affinity-dependent effects. Sci Transl Med. 2019;11:eaao5563. 10.1126/scitranslmed.aao5563 31043571

[49] Rossmann MP, Dubois SM, Agarwal S, Zon LI. Mitochondrial function in development and disease. Dis Model Mech. 2021;14:dmm048912. 10.1242/dmm.048912 PMC8214736 34114603

[50] Brand MD, Orr AL, Perevoshchikova IV, Quinlan CL. The role of mitochondrial function and cellular bioenergetics in ageing and disease. Br J Dermatol. 2013;169:1–8. 10.1111/bjd.12208 PMC4321783 23786614

[51] Spinelli JB, Haigis MC. The multifaceted contributions of mitochondria to cellular metabolism. Nat Cell Biol. 2018;20:745–54. 10.1038/s41556-018-0124-1 PMC6541229 29950572

[52] De Biasi S, Simone AM, Bianchini E, Lo Tartaro D, Pecorini S, Nasi M, et al. Mitochondrial functionality and metabolism in T cells from progressive multiple sclerosis patients. Eur J Immunol. 2019;49:2204–21. 10.1002/eji.201948223 31373677

[53] Ng X, Sadeghian M, Heales S, Hargreaves IP. Assessment of Mitochondrial Dysfunction in Experimental Autoimmune Encephalomyelitis (EAE) Models of Multiple Sclerosis. Int J Mol Sci. 2019;20:4975. 10.3390/ijms20204975 PMC6829485 31600882

[54] Julià E, Edo MC, Horga A, Montalban X, Comabella M. Differential susceptibility to apoptosis of CD4+T cells expressing CCR5 and CXCR3 in patients with MS. Clin Immunol. 2009;133:364–74. 10.1016/j.clim.2009.08.012 19740704

[55] Sharief MK, Douglas M, Noori M, Semra YK. The expression of pro- and anti-apoptosis Bcl-2 family proteins in lymphocytes from patients with multiple sclerosis. J Neuroimmunol. 2002;125:155–62. 10.1016/s0165-5728(02)00024-3 11960652

[56] Ho PP, Kanter JL, Johnson AM, Srinagesh HK, Chang EJ, Purdy TM, et al. Identification of naturally occurring fatty acids of the myelin sheath that resolve neuroinflammation. Sci Transl Med. 2012;4:137ra73. 10.1126/scitranslmed.3003831 PMC3953135 22674551

[57] Duscha A, Gisevius B, Hirschberg S, Yissachar N, Stangl GI, Dawin E, et al. Propionic Acid Shapes the Multiple Sclerosis Disease Course by an Immunomodulatory Mechanism. Cell. 2020;180:1067–80.e16. 10.1016/j.cell.2020.02.035 32160527

[58] Dyczko A, Côrte-Real BF, Hamad I, Linker RA, Kleinewietfeld M. Teriflunomide Inhibits Human FOXP3(+) Regulatory T Cell Function by Interference With Mitochondrial Respiration. Eur J Immunol. 2025;55:e51260. 10.1002/eji.202451260 40879143

[59] Alissafi T, Kalafati L, Lazari M, Filia A, Kloukina I, Manifava M, et al. Mitochondrial Oxidative Damage Underlies Regulatory T Cell Defects in Autoimmunity. Cell Metab. 2020;32:591–604.e7. 10.1016/j.cmet.2020.07.001 PMC7611060 32738205

[60] Shin B, Benavides GA, Geng J, Koralov SB, Hu H, Darley-Usmar VM, et al. Mitochondrial Oxidative Phosphorylation Regulates the Fate Decision between Pathogenic Th17 and Regulatory T Cells. Cell Rep. 2020;30:1898–909.e4. 10.1016/j.celrep.2020.01.022 PMC9059282 32049019

[61] Almeida L, Dhillon-LaBrooy A, Castro CN, Adossa N, Carriche GM, Guderian M, et al. Ribosome-Targeting Antibiotics Impair T Cell Effector Function and Ameliorate Autoimmunity by Blocking Mitochondrial Protein Synthesis. Immunity. 2021;54:68–83.e6. 10.1016/j.immuni.2020.11.001 PMC7837214 33238133

[62] Pompura SL, Hafler DA. Dominguez-Villar M. Fatty Acid Metabolism and T Cells in Multiple Sclerosis. Front Immunol. 2022;13:869197. 10.3389/fimmu.2022.869197 PMC9116144 35603182

[63] Vergara D, D’Alessandro M, Rizzello A, De Riccardis L, Lunetti P, Del Boccio P, et al. A lipidomic approach to the study of human CD4(+) T lymphocytes in multiple sclerosis. BMC Neurosci. 2015;16:46. 10.1186/s12868-015-0183-1 PMC4513631 26205308

[64] Pompura SL, Wagner A, Kitz A, LaPerche J, Yosef N, Dominguez-Villar M, et al. Oleic acid restores suppressive defects in tissue-resident FOXP3 Tregs from patients with multiple sclerosis. J Clin Invest. 2021;131:e138519. 10.1172/JCI138519 PMC7810477 33170805

[65] Luu M, Pautz S, Kohl V, Singh R, Romero R, Lucas S, et al. The short-chain fatty acid pentanoate suppresses autoimmunity by modulating the metabolic-epigenetic crosstalk in lymphocytes. Nat Commun. 2019;10:760. 10.1038/s41467-019-08711-2 PMC6377655 30770822

[66] Haase S, Mäurer J, Duscha A, Lee DH, Balogh A, Gold R, et al. Propionic Acid Rescues High-Fat Diet Enhanced Immunopathology in Autoimmunity via Effects on Th17 Responses. Front Immunol. 2021;12:701626. 10.3389/fimmu.2021.701626 PMC8204048 34140958

[67] Rao E, Singh P, Li Y, Zhang Y, Chi YI, Suttles J, et al. Targeting epidermal fatty acid binding protein for treatment of experimental autoimmune encephalomyelitis. BMC Immunol. 2015;16:28. 10.1186/s12865-015-0091-2 PMC4427938 25962726

[68] Youssef S, Stüve O, Patarroyo JC, Ruiz PJ, Radosevich JL, Hur EM, et al. The HMG-CoA reductase inhibitor, atorvastatin, promotes a Th2 bias and reverses paralysis in central nervous system autoimmune disease. Nature. 2002;420:78–84. 10.1038/nature01158 12422218

[69] Sinha S, Boyden AW, Itani FR, Crawford MP, Karandikar NJ. CD8(+) T-Cells as Immune Regulators of Multiple Sclerosis. Front Immunol. 2015;6:619. 10.3389/fimmu.2015.00619 PMC4674574 26697014

[70] Wang PF, Jiang F, Zeng QM, Yin WF, Hu YZ, Li Q, et al. Mitochondrial and metabolic dysfunction of peripheral immune cells in multiple sclerosis. J Neuroinflammation. 2024;21:28. 10.1186/s12974-024-03016-8 PMC10799425 38243312

[71] Cao J, Liao S, Zeng F, Liao Q, Luo G, Zhou Y. Effects of altered glycolysis levels on CD8(+) T cell activation and function. Cell Death Dis. 2023;14:407. 10.1038/s41419-023-05937-3 PMC10329707 37422501

[72] Tilly G, Cadoux M, Garcia A, Morille J, Wiertlewski S, Pecqueur C, et al. Teriflunomide Treatment of Multiple Sclerosis Selectively Modulates CD8 Memory T Cells. Front Immunol. 2021;12:730342. 10.3389/fimmu.2021.730342 PMC8552527 34721394

[73] Liebmann M, Korn L, Janoschka C, Albrecht S, Lauks S, Herrmann AM, et al. Dimethyl fumarate treatment restrains the antioxidative capacity of T cells to control autoimmunity. Brain. 2021;144:3126–41. 10.1093/brain/awab307 PMC8634070 34849598

[74] Hauser SL, Bar-Or A, Comi G, Giovannoni G, Hartung HP, Hemmer B, et al. Ocrelizumab versus Interferon Beta-1a in Relapsing Multiple Sclerosis. N Engl J Med. 2017;376:221–34. 10.1056/NEJMoa1601277 28002679

[75] Genç K, Dona DL, Reder AT. Increased CD80(+) B cells in active multiple sclerosis and reversal by interferon beta-1b therapy. J Clin Invest. 1997;99:2664–71. 10.1172/JCI119455 PMC508112 9169496

[76] Mathias A, Perriard G, Canales M, Soneson C, Delorenzi M, Schluep M, et al. Increased ex vivo antigen presentation profile of B cells in multiple sclerosis. Mult Scler. 2017;23:802–9. 10.1177/1352458516664210 27503907

[77] Piancone F, Saresella M, Marventano I, La Rosa F, Zoppis M, Agostini S, et al. B Lymphocytes in Multiple Sclerosis: Bregs and BTLA/CD272 Expressing-CD19+ Lymphocytes Modulate Disease Severity. Sci Rep. 2016;6:29699. 10.1038/srep29699 PMC4944189 27412504

[78] Barr TA, Shen P, Brown S, Lampropoulou V, Roch T, Lawrie S, et al. B cell depletion therapy ameliorates autoimmune disease through ablation of IL-6-producing B cells. J Exp Med. 2012;209:1001–10. 10.1084/jem.20111675 PMC3348102 22547654

[79] Li R, Tang H, Burns JC, Hopkins BT, Le Coz C, Zhang B, et al. BTK inhibition limits B-cell-T-cell interaction through modulation of B-cell metabolism: implications for multiple sclerosis therapy. Acta Neuropathol. 2022;143:505–21. 10.1007/s00401-022-02411-w PMC8960592 35303161

[80] Kolln J, Ren HM, Da RR, Zhang Y, Spillner E, Olek M, et al. Triosephosphate isomerase- and glyceraldehyde-3-phosphate dehydrogenase-reactive autoantibodies in the cerebrospinal fluid of patients with multiple sclerosis. J Immunol. 2006;177:5652–8. 10.4049/jimmunol.177.8.5652 17015754

[81] Li R, Lei Y, Rezk A, Diego AE, Wang J, Feng H, et al. Oxidative phosphorylation regulates B cell effector cytokines and promotes inflammation in multiple sclerosis. Sci Immunol. 2024;9:eadk0865. 10.1126/sciimmunol.adk0865 38701189

[82] Mellado M, Martínez-Muñoz L, Cascio G, Lucas P, Pablos JL, Rodríguez-Frade JMT. Cell Migration in Rheumatoid Arthritis. Front Immunol. 2015;6:384. 10.3389/fimmu.2015.00384 PMC4515597 26284069

[83] Chemin K, Gerstner C, Malmström V. Effector Functions of CD4+T Cells at the Site of Local Autoimmune Inflammation-Lessons From Rheumatoid Arthritis. Front Immunol. 2019;10:353. 10.3389/fimmu.2019.00353 PMC6422991 30915067

[84] Jang S, Kwon EJ, Lee JJ. Rheumatoid Arthritis: Pathogenic Roles of Diverse Immune Cells. Int J Mol Sci. 2022;23:905. 10.3390/ijms23020905 PMC8780115 35055087

[85] Yang Z, Fujii H, Mohan SV, Goronzy JJ, Weyand CM. Phosphofructokinase deficiency impairs ATP generation, autophagy, and redox balance in rheumatoid arthritis T cells. J Exp Med. 2013;210:2119–34. 10.1084/jem.20130252 PMC3782046 24043759

[86] Weyand CM, Goronzy JJ. Immunometabolism in the development of rheumatoid arthritis. Immunol Rev. 2020;294:177–87. 10.1111/imr.12838 PMC7047523 31984519

[87] Yang Z, Shen Y, Oishi H, Matteson EL, Tian L, Goronzy JJ, et al. Restoring oxidant signaling suppresses proarthritogenic T cell effector functions in rheumatoid arthritis. Sci Transl Med. 2016;8:331ra38. 10.1126/scitranslmed.aad7151 PMC5074090 27009267

[88] Okano T, Saegusa J, Nishimura K, Takahashi S, Sendo S, Ueda Y, et al. 3-bromopyruvate ameliorate autoimmune arthritis by modulating Th17/Treg cell differentiation and suppressing dendritic cell activation. Sci Rep. 2017;7:42412. 10.1038/srep42412 PMC5301239 28186160

[89] Wen Z, Jin K, Shen Y, Yang Z, Li Y, Wu B, et al. N-myristoyltransferase deficiency impairs activation of kinase AMPK and promotes synovial tissue inflammation. Nat Immunol. 2019;20:313–25. 10.1038/s41590-018-0296-7 PMC6396296 30718913

[90] Bai Z, Yang S, Ren J, Zhang C, Chen X, Huang H, et al. Exploring the differential functions of circulating follicular helper T and peripheral helper T cells in rheumatoid arthritis based on metabolism patterns. Front Immunol. 2025;16:1608675. 10.3389/fimmu.2025.1608675 PMC12209185 40599793

[91] Yang EJ, Lee J, Lee SY, Kim EK, Moon YM, Jung YO, et al. EGCG attenuates autoimmune arthritis by inhibition of STAT3 and HIF-1α with Th17/Treg control. PLoS One. 2014;9:e86062. 10.1371/journal.pone.0086062 PMC3928092 24558360

[92] Pucino V, Certo M, Bulusu V, Cucchi D, Goldmann K, Pontarini E, et al. Lactate Buildup at the Site of Chronic Inflammation Promotes Disease by Inducing CD4(+) T Cell Metabolic Rewiring. Cell Metab. 2019;30:1055–74.e8. 10.1016/j.cmet.2019.10.004 PMC6899510 31708446

[93] Wu B, Qiu J, Zhao TV, Wang Y, Maeda T, Goronzy IN, et al. Succinyl-CoA Ligase Deficiency in Pro-inflammatory and Tissue-Invasive T Cells. Cell Metab. 2020;32:967–80.e5. 10.1016/j.cmet.2020.10.025 PMC7755381 33264602

[94] Shen Y, Wen Z, Li Y, Matteson EL, Hong J, Goronzy JJ, et al. Metabolic control of the scaffold protein TKS5 in tissue-invasive, proinflammatory T cells. Nat Immunol. 2017;18:1025–34. 10.1038/ni.3808 PMC5568495 28737753

[95] Han J, Zhou Z, Wang H, Chen Y, Li W, Dai M, et al. Dysfunctional glycolysis-UCP2-fatty acid oxidation promotes CTLA4(int)FOXP3(int) regulatory T-cell production in rheumatoid arthritis. Mol Med. 2025;31:310. 10.1186/s10020-025-01372-6 PMC12512393 41068616

[96] Li Y, Shen Y, Hohensinner P, Ju J, Wen Z, Goodman SB, et al. Deficient Activity of the Nuclease MRE11A Induces T Cell Aging and Promotes Arthritogenic Effector Functions in Patients with Rheumatoid Arthritis. Immunity. 2016;45:903–16. 10.1016/j.immuni.2016.09.013 PMC5123765 27742546

[97] Wu B, Zhao TV, Jin K, Hu Z, Abdel MP, Warrington KJ, et al. Mitochondrial aspartate regulates TNF biogenesis and autoimmune tissue inflammation. Nat Immunol. 2021;22:1551–62. 10.1038/s41590-021-01065-2 PMC8756813 34811544

[98] Moon JS, Younis S, Ramadoss NS, Iyer R, Sheth K, Sharpe O, et al. Cytotoxic CD8(+) T cells target citrullinated antigens in rheumatoid arthritis. Nat Commun. 2023;14:319. 10.1038/s41467-022-35264-8 PMC9852471 36658110

[99] Carvalheiro H, da Silva JA, Souto-Carneiro MM. Potential roles for CD8(+) T cells in rheumatoid arthritis. Autoimmun Rev. 2013;12:401–9. 10.1016/j.autrev.2012.07.011 22841983

[100] Souto-Carneiro MM, Klika KD, Abreu MT, Meyer AP, Saffrich R, Sandhoff R, et al. Effect of Increased Lactate Dehydrogenase A Activity and Aerobic Glycolysis on the Proinflammatory Profile of Autoimmune CD8+T Cells in Rheumatoid Arthritis. Arthritis Rheumatol. 2020;72:2050–64. 10.1002/art.41420 32602217

[101] Kraus FV, Keck S, Klika KD, Graf J, Carvalho RA, Lorenz HM, et al. Reduction of Proinflammatory Effector Functions Through Remodeling of Fatty Acid Metabolism in CD8+T Cells From Rheumatoid Arthritis Patients. Arthritis Rheumatol. 2023;75:1098–109. 10.1002/art.42456 36704915

[102] Silverman GJ, Carson DA. Roles of B cells in rheumatoid arthritis. Arthritis Res Ther. 2003;5:S1–6. 10.1186/ar1010 PMC2833442 15180890

[103] Bugatti S, Vitolo B, Caporali R, Montecucco C, Manzo A. B cells in rheumatoid arthritis: from pathogenic players to disease biomarkers. Biomed Res Int. 2014;2014:681678. 10.1155/2014/681678 PMC4022166 24877127

[104] Bombardieri M, Lewis M, Pitzalis C. Ectopic lymphoid neogenesis in rheumatic autoimmune diseases. Nat Rev Rheumatol. 2017;13:141–54. 10.1038/nrrheum.2016.217 28202919

[105] Tang Y, Bai Z, Qi J, Lu Z, Ahmad, Wang G, et al. Altered peripheral B lymphocyte homeostasis and functions mediated by IL-27 via activating the mammalian target of rapamycin signaling pathway in patients with rheumatoid arthritis. Clin Exp Immunol. 2021;206:354–65. 10.1111/cei.13663 PMC8561691 34558072

[106] Floudas A, Neto N, Marzaioli V, Murray K, Moran B, Monaghan MG, et al. Pathogenic, glycolytic PD-1+B cells accumulate in the hypoxic RA joint. JCI Insight. 2020;5:e139032. 10.1172/jci.insight.139032 PMC7710281 33148884

[107] Abboud G, Choi SC, Kanda N, Zeumer-Spataro L, Roopenian DC, Morel L. Inhibition of Glycolysis Reduces Disease Severity in an Autoimmune Model of Rheumatoid Arthritis. Front Immunol. 2018;9:1973. 10.3389/fimmu.2018.01973 PMC6130222 30233578

[108] Khmaladze I, Saxena A, Nandakumar KS, Holmdahl R. B-cell epitope spreading and inflammation in a mouse model of arthritis is associated with a deficiency in reactive oxygen species production. Eur J Immunol. 2015;45:2243–51. 10.1002/eji.201545518 25989352

[109] Weyand CM, Goronzy JJ. Immunometabolism in early and late stages of rheumatoid arthritis. Nat Rev Rheumatol. 2017;13:291–301. 10.1038/nrrheum.2017.49 PMC6820517 28360422

[110] Shao L, Fujii H, Colmegna I, Oishi H, Goronzy JJ, Weyand CM. Deficiency of the DNA repair enzyme ATM in rheumatoid arthritis. J Exp Med. 2009;206:1435–49. 10.1084/jem.20082251 PMC2715066 19451263

[111] Lehuen A, Diana J, Zaccone P, Cooke A. Immune cell crosstalk in type 1 diabetes. Nat Rev Immunol. 2010;10:501–13. 10.1038/nri2787 20577267

[112] Pugliese A. Autoreactive T cells in type 1 diabetes. J Clin Invest. 2017;127:2881–91. 10.1172/JCI94549 PMC5531393 28762987

[113] Peters LD, Yeh WI, Arnoletti JM, Brown ME, Posgai AL, Mathews CE, et al. Modeling cell-mediated immunity in human type 1 diabetes by engineering autoreactive CD8(+) T cells. Front Immunol. 2023;14:1142648. 10.3389/fimmu.2023.1142648 PMC10262917 37325626

[114] Chatenoud L, Marquet C, Valette F, Scott L, Quan J, Bu CH, et al. Modulation of autoimmune diabetes by N-ethyl-N-nitrosourea- induced mutations in non-obese diabetic mice. Dis Model Mech. 2022;15:dmm049484. 10.1242/dmm.049484 PMC9178510 35502705

[115] Padgett LE, Broniowska KA, Hansen PA, Corbett JA, Tse HM. The role of reactive oxygen species and proinflammatory cytokines in type 1 diabetes pathogenesis. Ann N Y Acad Sci. 2013;1281:16–35. 10.1111/j.1749-6632.2012.06826.x PMC3715103 23323860

[116] Reinert-Hartwall L, Honkanen J, Salo HM, Nieminen JK, Luopajärvi K, Härkönen T, et al. Th1/Th17 plasticity is a marker of advanced β cell autoimmunity and impaired glucose tolerance in humans. J Immunol. 2015;194:68–75. 10.4049/jimmunol.1401653 PMC4273995 25480564

[117] Yi Z, Li L, Garland A, He Q, Wang H, Katz JD, et al. IFN-γ receptor deficiency prevents diabetes induction by diabetogenic CD4+, but not CD8+, T cells. Eur J Immunol. 2012;42:2010–8. 10.1002/eji.201242374 PMC3883988 22865049

[118] Abdel-Moneim A, Bakery HH, Allam G. The potential pathogenic role of IL-17/Th17 cells in both type 1 and type 2 diabetes mellitus. Biomed Pharmacother. 2018;101:287–92. 10.1016/j.biopha.2018.02.103 29499402

[119] Martins CP, New LA, O’Connor EC, Previte DM, Cargill KR, Tse IL, et al. Glycolysis Inhibition Induces Functional and Metabolic Exhaustion of CD4(+) T Cells in Type 1 Diabetes. Front Immunol. 2021;12:669456. 10.3389/fimmu.2021.669456 PMC8216385 34163475

[120] Battaglia M, Stabilini A, Migliavacca B, Horejs-Hoeck J, Kaupper T, Roncarolo MG. Rapamycin promotes expansion of functional CD4+CD25+FOXP3+ regulatory T cells of both healthy subjects and type 1 diabetic patients. J Immunol. 2006;177:8338–47. 10.4049/jimmunol.177.12.8338 17142730

[121] Long SA, Rieck M, Sanda S, Bollyky JB, Samuels PL, Goland R, et al. Rapamycin/IL-2 combination therapy in patients with type 1 diabetes augments Tregs yet transiently impairs β-cell function. Diabetes. 2012;61:2340–8. 10.2337/db12-0049 PMC3425404 22721971

[122] Duan W, Ding Y, Yu X, Ma D, Yang B, Li Y, et al. Metformin mitigates autoimmune insulitis by inhibiting Th1 and Th17 responses while promoting Treg production. Am J Transl Res. 2019;11:2393–402. PMC6511786 31105845

[123] Zhang M, Zhou Y, Xie Z, Luo S, Zhou Z, Huang J, et al. New Developments in T Cell Immunometabolism and Therapeutic Implications for Type 1 Diabetes. Front Endocrinol (Lausanne). 2022;13:914136. 10.3389/fendo.2022.914136 PMC9226440 35757405

[124] Chen J, Stimpson SE, Fernandez-Bueno GA, Mathews CE. Mitochondrial Reactive Oxygen Species and Type 1 Diabetes. Antioxid Redox Signal. 2018;29:1361–72. 10.1089/ars.2017.7346 PMC6166689 29295631

[125] Chen XL, Serrano D, Mayhue M, Wieden HJ, Stankova J, Boulay G, et al. GTPase of the immune-associated nucleotide-binding protein 5 (GIMAP5) regulates calcium influx in T-lymphocytes by promoting mitochondrial calcium accumulation. Biochem J. 2013;449:353–64. 10.1042/BJ20120516 23098229

[126] Chen J, Chernatynskaya AV, Li JW, Kimbrell MR, Cassidy RJ, Perry DJ, et al. T cells display mitochondria hyperpolarization in human type 1 diabetes. Sci Rep. 2017;7:10835. 10.1038/s41598-017-11056-9 PMC5589742 28883439

[127] Jia L, Zhang L, Liu M, Ji H, Wen Z, Wang C. Mitochondrial Control for Healthy and Autoimmune T Cells. Cells. 2023;12:1800. 10.3390/cells12131800 PMC10340733 37443834

[128] Jeong H, Lee B, Han SJ, Sohn DH. Glucose metabolic reprogramming in autoimmune diseases. Anim Cells Syst (Seoul). 2023;27:149–58. 10.1080/19768354.2023.2234986 PMC10351453 37465289

[129] Coudriet GM, Delmastro-Greenwood MM, Previte DM, Marré ML, O’Connor EC, Novak EA, et al. Treatment with a Catalytic Superoxide Dismutase (SOD) Mimetic Improves Liver Steatosis, Insulin Sensitivity, and Inflammation in Obesity-Induced Type 2 Diabetes. Antioxidants (Basel). 2017;6:85. 10.3390/antiox6040085 PMC5745495 29104232

[130] Miki A, Ricordi C, Sakuma Y, Yamamoto T, Misawa R, Mita A, et al. Divergent antioxidant capacity of human islet cell subsets: A potential cause of beta-cell vulnerability in diabetes and islet transplantation. PLoS One. 2018;13:e0196570. 10.1371/journal.pone.0196570 PMC5933778 29723228

[131] Delmastro-Greenwood MM, Votyakova T, Goetzman E, Marre ML, Previte DM, Tovmasyan A, et al. Mn porphyrin regulation of aerobic glycolysis: implications on the activation of diabetogenic immune cells. Antioxid Redox Signal. 2013;19:1902–15. 10.1089/ars.2012.5167 PMC3931434 23682840

[132] Piganelli JD, Flores SC, Cruz C, Koepp J, Batinic-Haberle I, Crapo J, et al. A metalloporphyrin-based superoxide dismutase mimic inhibits adoptive transfer of autoimmune diabetes by a diabetogenic T-cell clone. Diabetes. 2002;51:347–55. 10.2337/diabetes.51.2.347 11812741

[133] Kang S, Liu L, Wang T, Cannon M, Lin P, Fan TW, et al. GAB functions as a bioenergetic and signalling gatekeeper to control T cell inflammation. Nat Metab. 2022;4:1322–35. 10.1038/s42255-022-00638-1 PMC9584824 36192601

[134] Tian J, Lu Y, Zhang H, Chau CH, Dang HN, Kaufman DL. Gamma-aminobutyric acid inhibits T cell autoimmunity and the development of inflammatory responses in a mouse type 1 diabetes model. J Immunol. 2004;173:5298–304. 10.4049/jimmunol.173.8.5298 15470076

[135] Jones AI, Coleman EL, Husni NR, Deeney JT, Raval F, Steenkamp D, et al. Type 1 diabetes alters lipid handling and metabolism in human fibroblasts and peripheral blood mononuclear cells. PLoS One. 2017;12:e0188474. 10.1371/journal.pone.0188474 PMC5714353 29206239

[136] McKenzie MD, Dudek NL, Mariana L, Chong MM, Trapani JA, Kay TW, et al. Perforin and Fas induced by IFNgamma and TNFalpha mediate beta cell death by OT-I CTL. Int Immunol. 2006;18:837–46. 10.1093/intimm/dxl020 16574667

[137] Kasmani MY, Ciecko AE, Brown AK, Petrova G, Gorski J, Chen YG, et al. Autoreactive CD8 T cells in NOD mice exhibit phenotypic heterogeneity but restricted TCR gene usage. Life Sci Alliance. 2022;5:e202201503. 10.26508/lsa.202201503 PMC9170949 35667687

[138] Garyu JW, Uduman M, Stewart A, Rui J, Deng S, Shenson J, et al. Characterization of Diabetogenic CD8+T Cells: IMMUNE THERAPY WITH METABOLIC BLOCKADE. J Biol Chem. 2016;291:11230–40. 10.1074/jbc.M115.713362 PMC4900270 26994137

[139] Sklavos MM, Tse HM, Piganelli JD. Redox modulation inhibits CD8 T cell effector function. Free Radic Biol Med. 2008;45:1477–86. 10.1016/j.freeradbiomed.2008.08.023 18805480

[140] Wilson CS, Moore DJB Cell Metabolism: An Understudied Opportunity to Improve Immune Therapy in Autoimmune Type 1 Diabetes. Immunometabolism. 2020;2.

[141] Smith MJ, Simmons KM, Cambier JC. B cells in type 1 diabetes mellitus and diabetic kidney disease. Nat Rev Nephrol. 2017;13:712–20. 10.1038/nrneph.2017.138 PMC6733025 29038537

[142] Li Z, Zhao M, Li J, Luo W, Huang J, Huang G, et al. Elevated glucose metabolism driving pro-inflammatory response in B cells contributes to the progression of type 1 diabetes. Clin Immunol. 2023;255:109729. 10.1016/j.clim.2023.109729 37562723

[143] Zhu M, Li X, Zhao K, Huang Z, Zhao T. Altered glucose metabolism in B cells: Implications for the pathogenesis and treatment of autoimmune diseases. J Autoimmun. 2025;156:103472. 10.1016/j.jaut.2025.103472 40811884

[144] Conway H, Bernard J, Ramos R, Zahran S, Perez DL, Swar-Eldahab M, et al. Altered B cell metabolic pathways characterize type 1 diabetes progression. J Immunol. 2026;215:vkaf330. 10.1093/jimmun/vkaf330 PMC13008343 41454913

[145] Wilson CS, Elizer SK, Marshall AF, Stocks BT, Moore DJ. Regulation of B lymphocyte responses to Toll-like receptor ligand binding during diabetes prevention in non-obese diabetic (NOD) mice. J Diabetes. 2016;8:120–31. 10.1111/1753-0407.12263 PMC4598313 25564999

[146] Finlay DK, Rosenzweig E, Sinclair LV, Feijoo-Carnero C, Hukelmann JL, Rolf J, et al. PDK1 regulation of mTOR and hypoxia-inducible factor 1 integrate metabolism and migration of CD8+T cells. J Exp Med. 2012;209:2441–53. 10.1084/jem.20112607 PMC3526360 23183047

[147] Almogbel E, Rasheed N. Protein Mediated Oxidative Stress in Patients with Diabetes and its Associated Neuropathy: Correlation with Protein Carbonylation and Disease Activity Markers. J Clin Diagn Res. 2017;11:Bc21–bc5. 10.7860/JCDR/2017/23789.9417 PMC5376809 28384853

[148] Previte DM, O’Connor EC, Novak EA, Martins CP, Mollen KP, Piganelli JD. Reactive oxygen species are required for driving efficient and sustained aerobic glycolysis during CD4+T cell activation. PLoS One. 2017;12:e0175549. 10.1371/journal.pone.0175549 PMC5398529 28426686

[149] Hill H, Elksnis A, Lundkvist P, Ubhayasekera K, Bergquist J, Birnir B, et al. Endogenous Levels of Gamma Amino-Butyric Acid Are Correlated to Glutamic-Acid Decarboxylase Antibody Levels in Type 1 Diabetes. Biomedicines. 2021;10:91. 10.3390/biomedicines10010091 PMC8773285 35052771

[150] Morel L. Immunometabolism in systemic lupus erythematosus. Nat Rev Rheumatol. 2017;13:280–90. 10.1038/nrrheum.2017.43 28360423

[151] Lee DSW, Rojas OL, Gommerman JL. B cell depletion therapies in autoimmune disease: advances and mechanistic insights. Nat Rev Drug Discov. 2021;20:179–99. 10.1038/s41573-020-00092-2 PMC7737718 33324003

[152] Sharabi A, Tsokos GC. T cell metabolism: new insights in systemic lupus erythematosus pathogenesis and therapy. Nat Rev Rheumatol. 2020;16:100–12. 10.1038/s41584-019-0356-x 31949287

[153] Jin L, Ding M, Cui S, Yang L, Zhao J, He J, et al. Aberrant glucose metabolism drives dysfunction of CD4(+) T cells in systemic lupus erythematosus and disease flares. Cent Eur J Immunol. 2025;50:13–23. 10.5114/ceji.2025.149252 PMC12224248 40620656

[154] Liao HJ, Chu CL, Wang SC, Lee HY, Wu CS. Increased HIF-1α expression in T cells and associated with enhanced Th17 pathway in systemic lupus erythematosus. J Formos Med Assoc. 2022;121:2446–56. 10.1016/j.jfma.2022.05.003 35599104

[155] Kato H, Perl A. Mechanistic target of rapamycin complex 1 expands Th17 and IL-4+CD4-CD8- double-negative T cells and contracts regulatory T cells in systemic lupus erythematosus. J Immunol. 2014;192:4134–44. 10.4049/jimmunol.1301859 PMC3995867 24683191

[156] Gergely P Jr., Niland B, Gonchoroff N. Pullmann R, Jr., Phillips PE, Perl A. Persistent mitochondrial hyperpolarization, increased reactive oxygen intermediate production, and cytoplasmic alkalinization characterize altered IL-10 signaling in patients with systemic lupus erythematosus. J Immunol. 2002;169:1092–101. 10.4049/jimmunol.169.2.1092 PMC4020441 12097418

[157] Buskiewicz IA, Montgomery T, Yasewicz EC, Huber SA, Murphy MP, Hartley RC, et al. Reactive oxygen species induce virus-independent MAVS oligomerization in systemic lupus erythematosus. Sci Signal. 2016;9:ra115. 10.1126/scisignal.aaf1933 PMC5321043 27899525

[158] Doherty E, Oaks Z, Perl A. Increased mitochondrial electron transport chain activity at complex I is regulated by N-acetylcysteine in lymphocytes of patients with systemic lupus erythematosus. Antioxid Redox Signal. 2014;21:56–65. 10.1089/ars.2013.5702 PMC4048573 24673154

[159] Gergely P Jr., Grossman C, Niland B, Puskas F, Neupane H, Allam F, et al. Mitochondrial hyperpolarization and ATP depletion in patients with systemic lupus erythematosus. Arthritis Rheum. 2002;46:175–90. 10.1002/1529-0131(200201)46:1<175::AID-ART10015>3.0.CO;2-H PMC4020417 11817589

[160] Fernandez D, Bonilla E, Mirza N, Niland B, Perl A. Rapamycin reduces disease activity and normalizes T cell activation-induced calcium fluxing in patients with systemic lupus erythematosus. Arthritis Rheum. 2006;54:2983–8. 10.1002/art.22085 PMC4034146 16947529

[161] McDonald G, Deepak S, Miguel L, Hall CJ, Isenberg DA, Magee AI, et al. Normalizing glycosphingolipids restores function in CD4+T cells from lupus patients. J Clin Invest. 2014;124:712–24. 10.1172/JCI69571 PMC3904606 24463447

[162] Jury EC, Kabouridis PS, Flores-Borja F, Mageed RA, Isenberg DA. Altered lipid raft-associated signaling and ganglioside expression in T lymphocytes from patients with systemic lupus erythematosus. J Clin Invest. 2004;113:1176–87. 10.1172/JCI20345 PMC385405 15085197

[163] Wilson JJ, Wei J, Daamen AR, Sears JD, Bechtel E, Mayberry CL, et al. Glucose oxidation-dependent survival of activated B cells provides a putative novel therapeutic target for lupus treatment. iScience. 2023;26:107487. 10.1016/j.isci.2023.107487 PMC10448027 37636066

[164] Li W, Qu G, Choi SC, Cornaby C, Titov A, Kanda N, et al. Targeting T Cell Activation and Lupus Autoimmune Phenotypes by Inhibiting Glucose Transporters. Front Immunol. 2019;10:833. 10.3389/fimmu.2019.00833 PMC6478810 31057554

[165] Takeshima Y, Iwasaki Y, Nakano M, Narushima Y, Ota M, Nagafuchi Y, et al. Immune cell multiomics analysis reveals contribution of oxidative phosphorylation to B-cell functions and organ damage of lupus. Ann Rheum Dis. 2022;81:845–53. 10.1136/annrheumdis-2021-221464 35236659

[166] Zeng Q, Wang S, Li M, Wang S, Guo C, Ruan X, et al. Spleen fibroblastic reticular cell-derived acetylcholine promotes lipid metabolism to drive autoreactive B cell responses. Cell Metab. 2023;35:837–54.e8. 10.1016/j.cmet.2023.03.010 37019104

[167] Wang XY, Wei Y, Hu B, Liao Y, Wang X, Wan WH, et al. c-Myc-driven glycolysis polarizes functional regulatory B cells that trigger pathogenic inflammatory responses. Signal Transduct Target Ther. 2022;7:105. 10.1038/s41392-022-00948-6 PMC9013717 35430810

[168] Yin Y, Choi SC, Xu Z, Perry DJ, Seay H, Croker BP, et al. Normalization of CD4+T cell metabolism reverses lupus. Sci Transl Med. 2015;7:274ra18. 10.1126/scitranslmed.aaa0835 PMC5292723 25673763

[169] Delgoffe GM, Kole TP, Zheng Y, Zarek PE, Matthews KL, Xiao B, et al. The mTOR kinase differentially regulates effector and regulatory T cell lineage commitment. Immunity. 2009;30:832–44. 10.1016/j.immuni.2009.04.014 PMC2768135 19538929

[170] Lai ZW, Kelly R, Winans T, Marchena I, Shadakshari A, Yu J, et al. Sirolimus in patients with clinically active systemic lupus erythematosus resistant to, or intolerant of, conventional medications: a single-arm, open-label, phase 1/2 trial. Lancet. 2018;391:1186–96. 10.1016/S0140-6736(18)30485-9 PMC5891154 29551338

[171] Aderinto N, Abdulbasit MO, Tangmi ADE, Okesanya JO, Mubarak JM. Unveiling the growing significance of metabolism in modulating immune cell function: exploring mechanisms and implications; a review. Ann Med Surg (Lond). 2023;85:5511–22. 10.1097/MS9.0000000000001308 PMC10617839 37915697

[172] Haase S, Haghikia A, Wilck N, Müller DN, Linker RA. Impacts of microbiome metabolites on immune regulation and autoimmunity. Immunology. 2018;154:230–8. 10.1111/imm.12933 PMC5980218 29637999

[173] Siddiqui MT, Cresci GAM. The Immunomodulatory Functions of Butyrate. J Inflamm Res. 2021;14:6025–41. 10.2147/JIR.S300989 PMC8608412 34819742

[174] Zhou L, Zhang M, Wang Y, Dorfman RG, Liu H, Yu T, et al. Faecalibacterium prausnitzii Produces Butyrate to Maintain Th17/Treg Balance and to Ameliorate Colorectal Colitis by Inhibiting Histone Deacetylase 1. Inflamm Bowel Dis. 2018;24:1926–40. 10.1093/ibd/izy182 29796620

[175] Guo Y, Xiao Z, Wang Y, Yao W, Liao S, Yu B, et al. Sodium Butyrate Ameliorates Streptozotocin-Induced Type 1 Diabetes in Mice by Inhibiting the HMGB1 Expression. Front Endocrinol (Lausanne). 2018;9:630. 10.3389/fendo.2018.00630 PMC6209660 30410469

[176] Fujii J. Redox remodeling of central metabolism as a driving force for cellular protection, proliferation, differentiation, and dysfunction. Free Radic Res. 2024;58:606–29. 10.1080/10715762.2024.2407147 39316831

[177] Phégnon L, Pérochon J, Uttenweiler-Joseph S, Cahoreau E, Millard P, Létisse F. 6-Phosphogluconolactonase is critical for the efficient functioning of the pentose phosphate pathway. Febs j. 2024;291:4459–72. 10.1111/febs.17221 38982839

[178] Certo M, Tsai CH, Pucino V, Ho PC, Mauro C. Lactate modulation of immune responses in inflammatory versus tumour microenvironments. Nat Rev Immunol. 2021;21:151–61. 10.1038/s41577-020-0406-2 32839570

[179] Zhang S, Lv K, Liu Z, Zhao R, Li F. Fatty acid metabolism of immune cells: a new target of tumour immunotherapy. Cell Death Discov. 2024;10:39. 10.1038/s41420-024-01807-9 PMC10799907 38245525

[180] Huang H, Li G, He Y, Chen J, Yan J, Zhang Q, et al. Cellular succinate metabolism and signaling in inflammation: implications for therapeutic intervention. Front Immunol. 2024;15:1404441. 10.3389/fimmu.2024.1404441 PMC11200920 38933270

[181] Sun L, Yang X, Yuan Z, Wang H. Metabolic Reprogramming in Immune Response and Tissue Inflammation. Arterioscler Thromb Vasc Biol. 2020;40:1990–2001. 10.1161/ATVBAHA.120.314037 PMC7484156 32698683

[182] Humphrey SJ, James DE, Mann M. Protein Phosphorylation: A Major Switch Mechanism for Metabolic Regulation. Trends Endocrinol Metab. 2015;26:676–87. 10.1016/j.tem.2015.09.013 26498855

[183] Soliman GA. The role of mechanistic target of rapamycin (mTOR) complexes signaling in the immune responses. Nutrients. 2013;5:2231–57. 10.3390/nu5062231 PMC3725503 23783557

[184] Dimeloe S, Burgener AV, Grählert J, Hess C. T-cell metabolism governing activation, proliferation and differentiation; a modular view. Immunology. 2017;150:35–44. 10.1111/imm.12655 PMC5341500 27479920

[185] Liu Y, Zhang DT, Liu XG. mTOR signaling in T cell immunity and autoimmunity. Int Rev Immunol. 2015;34:50–66. 10.3109/08830185.2014.933957 25019278

[186] Tang YY, Wang DC, Wang YQ, Huang AF, Xu WD. Emerging role of hypoxia-inducible factor-1α in inflammatory autoimmune diseases: A comprehensive review. Front Immunol. 2022;13:1073971. 10.3389/fimmu.2022.1073971 PMC9905447 36761171

[187] Ji Z, He L, Regev A, Struhl K. Inflammatory regulatory network mediated by the joint action of NF-kB, STAT3, and AP-1 factors is involved in many human cancers. Proc Natl Acad Sci USA. 2019;116:9453–62. 10.1073/pnas.1821068116 PMC6511065 30910960

[188] Brescia C, Audia S, Pugliano A, Scaglione F, Iuliano R, Trapasso F, et al. Metabolic drives affecting Th17/Treg gene expression changes and differentiation: impact on immune-microenvironment regulation. Apmis. 2024;132:1026–45. 10.1111/apm.13378 38239016

[189] Masuko K. Glucose as a Potential Key to Fuel Inflammation in Rheumatoid Arthritis. Nutrients. 2022;14:2349. 10.3390/nu14112349 PMC9182926 35684149

[190] Chávez MD, Tse HM. Targeting Mitochondrial-Derived Reactive Oxygen Species in T Cell-Mediated Autoimmune Diseases. Front Immunol. 2021;12:703972. 10.3389/fimmu.2021.703972 PMC8281042 34276700

[191] Hubler MJ, Kennedy AJ. Role of lipids in the metabolism and activation of immune cells. J Nutr Biochem. 2016;34:1–7. 10.1016/j.jnutbio.2015.11.002 PMC5694687 27424223

[192] Song G, Lu Q, Fan H, Zhang X, Ge L, Tian R, et al. Inhibition of hexokinases holds potential as treatment strategy for rheumatoid arthritis. Arthritis Res Ther. 2019;21:87. 10.1186/s13075-019-1865-3 PMC6446273 30944034

[193] Prud’homme GJ, Glinka Y, Wang Q. Immunological GABAergic interactions and therapeutic applications in autoimmune diseases. Autoimmun Rev. 2015;14:1048–56. 10.1016/j.autrev.2015.07.011 26226414

